# Iron islands in the Amazon: investigating plant beta diversity of canga outcrops

**DOI:** 10.3897/phytokeys.165.54819

**Published:** 2020-10-28

**Authors:** Caroline Oliveira Andrino, Rafael Gomes Barbosa-Silva, Juliana Lovo, Pedro Lage Viana, Marcelo Freire Moro, Daniela Cristina Zappi

**Affiliations:** 1 Instituto Tecnológico Vale, Belém, Pará, Brazil Instituto Tecnológico Vale Belém Brazil; 2 Museu Paraense Emílio Goeldi, Coordenação Botânica, Belém, Pará, Brazil Museu Paraense Emílio Goeldi Belém Brazil; 3 Instituto de Ciências do Mar (Labomar), Universidade Federal do Ceará, Fortaleza, Ceará, Brazil Universidade Federal do Ceará Fortaleza Brazil

**Keywords:** campo rupestre, edaphic endemism, island-like habitats, Neotropical mountains, plant species diversity, rainforest, vascular plant survey

## Abstract

The world’s largest mineral iron province, Serra dos Carajás, is home to an open vegetation known as canga, found on top of isolated outcrops rising out of the Amazon rainforest. Over one thousand vascular plants species have been recorded in these canga sites, including 38 edaphic endemics. A new survey adds to our investigation of biogeographic relationships between sixteen canga outcrops and the effect of the distance between site pairs on the number of shared species, regional species turnover and species distribution patterns. Plant collecting expeditions to the westernmost site, the Serra de Campos of São Félix do Xingu (SFX), were carried out followed by the identification of all collected specimens and the creation of a species database, built to perform biogeographical analyses. Floristic relationships among the sites were investigated regarding their similarity, using multivariate analyses. The correlation between canga areas and species richness was tested, as well as the geographical distance between pairs of outcrops and their shared species. Vascular plants at SFX total 254 species including 17 edaphic endemics. All canga sites are grouped with 25% of minimum similarity, and the SFX falls within a large subgroup of outcrops. The total species number shared between site pairs does not change significantly with geographical distance but is positively correlated with the area of each outcrop. Meanwhile, shared endemic species numbers between site pairs decline when geographical distance increases, possibly imposed by the barrier of the rainforest. Our data suggest higher shared similarity between the largest and species-richest sites as opposed to geographically nearby sites, and provide useful insight for drafting conservation and compensation measures for canga locations. The size of the canga outcrops is associated to higher floristic diversity but connectivity among islands also plays a role in their similarity.

## Introduction

Mountaintops are often compared to sky-islands, as their vegetation is often distinct from the surrounding lowlands (Alves and Kolbek 2010; Barres et al. 2019). Montane habitats have been scrutinized due to their high species richness and complexity (Särkinen et al. 2012; Antonelli 2015; Kok et al. 2017), arousing scientific interest and have been featured since the first biogeographic studies (Humboldt 1805). In the Amazonian context, open vegetation predominates on exposed rocky surfaces on mountaintops, as opposed to the surrounding lowland rainforest. This vegetation may occur on isolated granite and gneiss inselbergs and quartzitic tepuis, usually above 900 m a.s.l. (Prance 1996; Riina et al. 2019), or over iron-ore conglomerates in the campo rupestre on canga (CRC), found between 600 and 800 m a.s.l. (Viana et al. 2016; Mota et al. 2018; Zappi et al. 2019). There are also island-like lowland ecosystems, such as white sand campinaranas, savannas, and low elevation granitic domes or inselbergs, associated with arenitic and often waterlogged soil in the Amazon region (Gröger and Huber 2007; Adeney et al. 2016; Costa et al. 2019; Henneron et al. 2019; Devecchi et al. 2020).

Canga is the lateritic duricrust that covers a supergene iron ore, with poorly developed soil and moderately hard rocks that are very resistant to erosion and permeable (Gagen et al. 2019). The iron-rich canga presents a series of restrictions to plant establishment, including shallow and rocky soils, high insolation levels, elevated temperatures at ground level, extreme water regime – waterlogged soil alternating with up to five months of drought, added to the presence of metals at potentially toxic concentrations (Schettini et al. 2018). The vegetation in the canga has specific strategies to survive in these stressful edaphic conditions (Gagen et al. 2019), and these conditions have favoured the diversification of edaphic endemic species that are exclusive to the CRC associated with the iron-rich substrate (Giulietti et al. 2019).

Species isolation caused by environmental conditions contrasting with the surrounding forests and associated with the mosaic of different geomorphological situations in the canga creates also an abundance of micro-habitats (Jacobi et al. 2007; Mota et al. 2015; Silva et al. 2020). It is known that such micro-habitats may be linked to multiple speciation events, and the occurrence of endemism (Bonatelli et al. 2014; Leal et al. 2016; Fiorini et al. 2019; Perrigo et al. 2019; Mota et al. 2020).

The first botanical studies on the iron islands of the Serra dos Carajás began in the late 1960s. However, the floristic knowledge was not synthetized and organized until the Flora of the canga of the Serra de Carajás (FCC) project was completed in 2018 (Viana et al. 2016; Mota et al. 2018). This recent flora increased the number of recorded species to 1042 vascular plants (Mota et al. 2018; Salino et al. 2018), and a number of species were confirmed as endemic to the local canga habitat, with 38 species occurring exclusively on this substrate in an area of occupancy of less than 150 km2 (Giulietti et al. 2019). In terms of phytophysiognomies, three major groups were defined by Mota et al. (2015) for Carajás: canga vegetation (scrub, bare slab, nodular canga and low forest grove), hydromorphic vegetation (bogs, temporary lagoons, permanent lakes, temporary streams, buriti palm lakes, swampy forest) and other associated forests (mostly at the edge of canga outcrops).

Due to historic reasons, collection efforts of the FCC project prioritized some areas of canga, while others still lack in-depth studies. For instance, a research in the canga of the Serra Arqueada (SA) in the municipality of Ourilândia do Norte has recently been completed (Fonseca-da-Silva et al. 2020), while the outcrops located within the recently created Parque Nacional dos Campos Ferruginosos (PNCF) are still in need of further investigation (Zappi et al. 2019). Giulietti et al. (2019) mentioned the existence of an interesting, isolated area of canga located c. 160 km southwest of the area studied by the FCC known as Serra de Campos, in the municipality of São Félix do Xingu (SFX).

This study aims to investigate plant distribution and biogeographical patterns that connect the island-like habitats of canga outcrops isolated within an Amazonian rainforest matrix. We evaluated species distribution in the different sites in order to observe whether canga vegetation has elevated levels of beta diversity and whether the flora of each outcrop will be more dissimilar to other outcrops as the geographical distance increases. We provided the first checklist of vascular plants growing on canga at the Serra de Campos of São Félix do Xingu (SFX), to add to the dataset we built to investigate the floristic relationship between canga areas, aiming to improve our understanding of the rich and diverse flora of the region.

## Methods

### Characterization of the overall study area

The CRC are found in the region of Carajás, located in the southeast part the State of Pará (Viana et al. 2016; Zappi et al. 2019), one of the largest mineral provinces in the world (Ab’saber 1986). At the Serra dos Carajás, the CRC appears atop a series of outcrops that form discontinuous island-like habitats of open, shrubby or grassy vegetation within a dense matrix of rainforest in the southeastern Amazon basin (Mota et al. 2018).

Most of the ferruginous island complex in the southeastern Amazon is within areas protected at different levels. The Serra Norte (SN1, SN2, SN3, SN4, SN5, SN6, SN7, SN8), the Serra Sul (S11A, S11B, S11CS11D) are located in the Floresta Nacional de Carajás, which is an area of sustainable use and thus subject to anthropogenic pressures, and iron ore mining currently occurs in areas SN4, SN5 and S11D. The Serra da Bocaina and Serra do Tarzan are the only fully protected areas, and are both inserted within the Parque Nacional dos Campos Ferruginosos (PNCF). However, the Serra Arqueada and Serra de Campos of São Félix do Xingu have no legal protection.

### Floristic list of Serra de Campos

**Figure 1.**
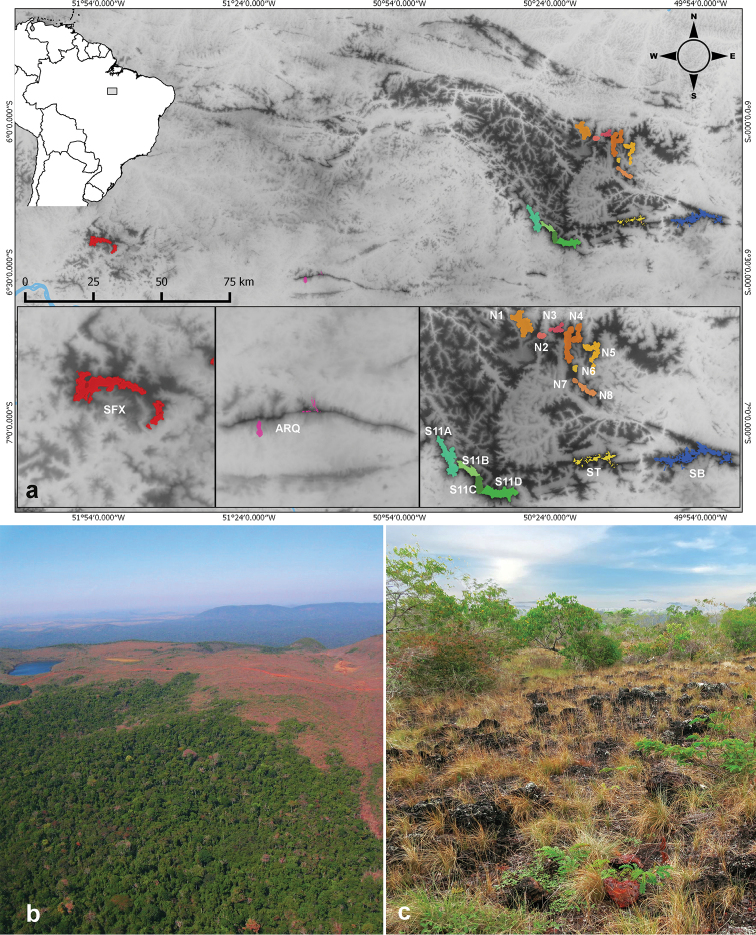
**a** Geographic location of the present study site at SFX and the other study areas from Carajás complex **b** aerial view of an island of *canga* vegetation surrounding by the rainforest (Photo: Leonardo Vianna) **c***Serra de Campos* of *São Félix do Xingu* (SFX) phytophysiognomy with shrubby and grassy vegetation.

The Serra de Campos (SFX) is a canga outcrop found in the municipality of São Félix do Xingu, southeastern Pará state, Brazilian Amazon. It represents the westernmost limit of the Serra dos Carajás, a complex of ferruginous highland outcrops that extends eastwards to the Municipality of Curionópolis, totalling 126 km2. The plateaus previously studied in the scope of the FCC project (Viana et al. 2016) are found in the Municipalities of Parauapebas (Serra Norte – SN1 to SN8), and Canaã dos Carajás (Serra Sul – S11, Serra do Tarzan – ST and Serra da Bocaina – SB). The SFX comprises two plateaus measuring c. 9 km2, distant about 1 km from each other, known as SFX1 and SFX2 (Fig. 1). The largest of the two plateaus, known as SFX2, extends for 8.5 km and covers an area of 7.6 km2, while SFX1 is 2.5 km long, measuring 1.4 km2. The plateaus are located at 6°23'41"S, 51°52'25"W, with altitudes ranging from 580 to 730 m. a.s.l. (Fig. 1). Distant about 80 km west from SA, the SFX can be accessed through the Municipality of São Felix do Xingu first by crossing the Rio Fresco then taking a road that goes through farmland, leading, after a steep climb, to the canga plateaus.

Botanical specimens from SFX deposited in herbaria prior to this study were located through an online search at the Herbarium of the Museu Paraense Emílio Goeldi (MG) and Herbário Ezechias Paulo Heringer (HEPH) (acronyms according to Thiers, continuously updated). Prior to our expeditions, specimens at MG were collected in the 1990´s by João Batista Fernandes da Silva and include the type of *Mimosa dasilvae* A.S.L. Silva & Secco and several gatherings of Orchidaceae, while HEPH currently holds collections made by Annajulia Elizabeth Heringer Salles and J.B.F. Silva in 2001. All materials available in these collections were analyzed and included in this study.

Four plant collecting expeditions were carried out between 2016 and 2019 (May 2016, April 2017, March 2018, October 2019), aiming to collect fertile material of all vascular species. Collecting method followed Filgueiras et al. (1994) with random walks covering the accessible parts of both plateaus, attempting to stop every 1 km to sample the vegetation and collect fertile specimens. We aimed to visit diverse vegetation types, including open canga slabs, nodular canga, canga scrub, palm swamps (buritizais) and temporary lagoons (Mota et al. 2015)

The samples collected were identified to species by comparing their macroscopic and microscopic morphological features with available bibliography, against herbarium collections (physically and on-line) and also consulting key family specialists. Voucher specimens were deposited at MG. Only one collection number per taxon is cited in the present floristic list. A full specimen list is provided in supplement S1. Species names follow Flora do Brasil online (Flora do Brasil under construction), family delimitation followed APG IV (Angiosperm Phylogeny Group 2016) and author abbreviations follow IPNI (2019).

### Database of the distribution of the flora of Serra dos Carajás

Seed plant species distribution data were assembled from the FCC project (Mota et al. 2018), with the compilation of a database comprising 3228 occurrences of 823 species (Zappi et al. 2019). The updates included 23 recent new occurrences for SN1, SN4, SN5, SN7, S11D, and the Serra da Bocaina based on recently collected herbarium material; 149 species for SA (Fonseca-da-Silva et al. 2020); and the newly prepared dataset of SFX. The assembled database comprises 909 seed plant species recorded in CRC at the Carajás complex, including 16 sites (SN1, SN2, SN3, SN4, SN5, SN6, SN7, SN8, S11A, S11B, S11C, S11D, ST, SB, SA and SFX). For the purpose of our analyses, exotic, invasive and weedy species were removed from the dataset as identified in (Giulietti et al. 2018), resulting in 893 species analysed. The code assigned for each site is found in Table 2.

### Biogeographical analyses of the flora of canga sites in the Carajás complex

To perform the biogeographical analysis of the CRC of the Carajás complex, the species database was used to investigate the floristic similarity and shared endemicity between different mountaintops across canga sites. Invasive exotic species recorded in each site were excluded from this analysis, as well as specimens with imprecise identification, Lycophytes, and Monilophytes. Floristic similarity between sites was calculated using a presence-absence Matrix (S2, Suppl. material 1) to perform multivariate analysis using ordination and group multivariate methods using the Vegan package in R (Oksanen et al. 2010). We constructed a matrix showing the presence of each species in each site and subjected it to ordination and grouping analyses using a Non-metric Multidimensional Scaling (NMDS) and Unweighted Pair Group Method with Arithmetic mean (UPGMA), respectively. Both analyses used Sorensen (Bray-Curtis) index (Legendre and Legendre 2012) to reflect beta diversity between sites.

To investigate the floristic richness of sites in relation to the size of each outcrop we used the species count for each canga outcrop and, employing GIS, we calculated the area of each outcrop in square kilometres. A linear model of the recorded richness versus area of each outcrop using the ‘glm’ function with Gaussian model was prepared in R. Because the outcrops were subjected to a large collecting effort during the ‘Flora of Carajás’ Project, we assumed that they were adequately sampled. We also evaluated whether the total number of species and of endemic species shared between sites were significantly related with the geographical distance between them. We computed the centroid of each outcrop using GIS and calculated the geographical distance between the centroids of all outcrop pairs. We tested the normality of the residuals of the models with the Shapiro-Wilk test to see whether the residuals significantly departed from normality. If these did not significantly differ from normality, we accepted the p value of the model. If the residuals differed from normality, we analysed the data using non parametric Spearman’s correlation to evaluate if the correlation was significant.

## Results

### Plant species in canga vegetation at Serra de Campos

**Figure 2.**
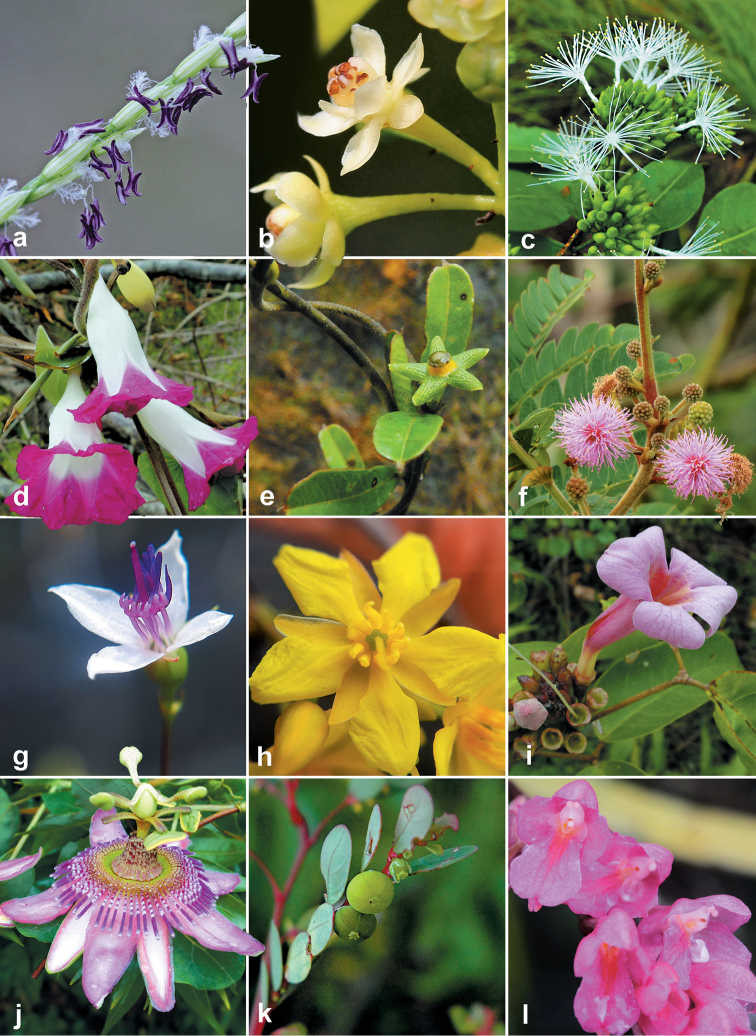
Representative species of canga in new dataset, SFX**a***Axonopus longispicus* (Döll) Kuhlm **b**Dicypellium aff. caryophyllaceum (Mart.) Nees **c***Inga heterophylla* Willd **d***Ipomoea decora* Meisn **e***Matelea microphylla* Morillo **f***Mimosa dasilvae* A.S.L. Silva & Secco **g***Nepsera aquatica* (Aubl.) Naudin **h***Ouratea cearensis* (Tiegh.) Sastre & Offroy **i***Pachyptera incarnata* (Aubl.) Francisco & L.G. Lohmann **j***Passifora picturata* Ker Gawl. **k***Phyllanthus minutulus* Mull.Arg. **l***Rodriguezia lanceolata* Ruiz & Pav.

This study recorded a total of 254 species, of which 248 are seed plants, five ferns and one lycophyte in the SFX (Table 1). The richest families recorded are Fabaceae (22 species), Poaceae (21 spp.), Cyperaceae (15 spp.), Orchidaceae (12 spp.) and Rubiaceae (12 spp.). The five richest genera are *Mimosa* (Fabaceae), with 5 species, *Cyperus* and *Rhynchospora* (Cyperaceae), with 4 species each, and *Borreria* (Rubiaceae) and *Aechmea* (Bromeliaceae), with 3 species each. Thirty-seven species are new records for the CRC of the *Carajás* complex. From these new records, seven belong to the family Orchidaceae, five are new records of Fabaceae, three Annonaceae, and three Sapindaceae. A yet undescribed species of Lauraceae was found in SFX, belonging to the genus *Dicypellium* (Dicypellium aff. caryophyllaceum (Mart.) Nees – PLV 6100, Table 1; Fig. 2).

**Table 1.** Vascular plant species from Serra de Campos of São Félix do Xingu (SFX), discriminated by novelties for Flora of the canga of Carajás according to Mota et al. (2018) and Fonseca-da-Silva et al. (2020) endemism in canga outcrops according to Giulietti et al. (2019); endemism in Serra de Campos, and life form and voucher information for each species. Collectors: AHS: Anajulia Heringer Salles; BF: Bruno Fernandes Falcão; COA: Caroline Oliveira Andrino; DCZ: Daniela Cristina Zappi; JBFS: João Batista da Silva; MN: Matheus Nogueira; MP: Mayara Pastore; PLV: Pedro Lage Viana. *Invasive exotic species.

Taxa	New for Carajás Flora	Endemic canga	Endemic SFX	Life form	Voucher
**Lycophyte**					
** Selaginellaceae **					
*Selaginella radiata* (Aubl.) Spring.				Herb	DCZ 4055
**Monilophytes**					
** Dennstaedtiaceae **					
*Pteridium arachnoideum* (Kauf.) Maxon				Herb	DCZ 4002
** Polypodiaceae **					
*Microgramma persicariifolia* (Schrad.) C.Presl				Herb	DCZ 4066
*Pleopeltis polypodioides* (L.) Andrews & Windham				Herb	DCZ 3922
*Serpocaulon triseriale* (Sw.) A.R.Sm.				Herb	DCZ 4037
** Pteridaceae **					
*Doryopteris collina* (Raddi) J.Sm.				Herb	DCZ 4040
**Spermathophytes**					
** Acanthaceae **					
*Justicia birae* A.S.Reis, F.A.Silva, A.Gil & Kameyama				Herb	MP 600
** Alismataceae **					
*Helanthium tenellum* (Mart. ex Schult & Schult.f.) Britton				Herb	MP 613
*Limnocharis flava* (L.) Buchenau	X			Herb	PLV 6149
** Anacardiaceae **					
*Anacardium occidentale* L.				Treelet	DCZ 3923
*Spondias mombin* L.	X			Treelet	DCZ 3921
** Annonaceae **					
*Annona sericea* Dunal	X			Shrub	DCZ 4051
*Annona exsucca* DC.				Tree	COA 658
*Guatteria procera* R.E.Fr.	X			Tree	DCZ 4050
*Xylopia aromatica* (Lam.) Mart.				Treelet	DCZ 3970
** Apocynaceae **					
Himatanthus cf. articulatus (Vahl) Woodson				Tree	COA 676
*Mandevilla scabra* (Hoffmanns. ex Roem. & Schult.) K. Schum.				Liana	DCZ 3880
*Mandevilla tenuifolia* (J.C. Mikan) Woodson				Herb	DCZ 3885
*Matelea microphylla* Morillo		X		Herb	DCZ 3942
*Tabernaemontana flavicans* Willd. ex Roem. & Schult.				Treelet	COA 613
*Tabernaemontana macrocalyx* Müll. Arg.				Treelet	COA 605
** Araceae **					
*Anthurium gracile* (Rudge) Lindl.				Herb	DCZ 5017
*Anthurium* sp.1		X		Herb	DCZ 3898
** Arecaceae **					
*Mauritia flexuosa* Mart.				Palm	DCZ 3961
*Mauritiella armata* (Mart.) Burret				Palm	DCZ 3960
*Oenocarpus distichus* Mart.				Palm	DCZ 3948
*Syagrus cocoides* Mart.				Palm	DCZ 3892
** Asteraceae **					
*Emilia fosbergii* Nicolson				Herb	DCZ 4046
*Ichthyothere terminalis* (Spreng.) S.F. Blake				Shrub	DCZ 3868
*Monogereion carajensis* G.M. Barroso & R.M. King		X		Herb	DCZ 3861
*Riencourtia pedunculosa* (Rich.) Pruski				Herb	DCZ 3924
*Tilesia baccata* (L.f.) Pruski				Herb	DCZ 3980
*Unxia camphorata* L.f.				Herb	DCZ 3941
** Begoniaceae **					
*Begonia humilis* Dryand				Herb	DCZ 3973
** Bignoniaceae **					
*Adenocalymma schomburgkii* (DC.) L.G.Lohmann				Liana	COA 611
*Amphilophium mansoanum* (DC.) L.G.Lohmann				Liana	DCZ 4025
*Anemopaegma carajasense* A.H. Gentry ex Firetti-Leggieri & L.G. Lohmann		X		Shrub	DCZ 3914
*Anemopaegma longipetiolatum* Sprague				Liana	DCZ 3867
*Jacaranda ulei* Bureau & K.Schum.				Shrub	DCZ 3945
*Pachyptera incarnata* (Aubl.) Francisco & L.G. Lohmann				Liana	DCZ 4061
*Pleonotoma melioides* (S.Moore) A.H.Gentry				Liana	COA 638
*Pleonotoma orientali*s Sandwith				Liana	DCZ 3883
** Bixaceae **					
*Cochlospermum orinocense* (Kunth) Steud.				Treelet	DCZ 3875
** Boraginaceae **					
*Cordia nodosa* Lam.				Tree	COA 641
** Bromeliaceae **					
*Aechmea castelnavii* Baker				Herb	COA 670
*Aechmea mertensii* (G.Mey.) Schult. & Schult.f.				Herb	COA 673
*Aechmea tocantina* Baker				Herb	AHS 2194
*Ananas ananassoides* (Baker) L.B. Sm.				Herb	DCZ 3891
*Dyckia duckei* L.B.Sm.				Herb	DCZ 3872
*Tillandsia adpressiflora* Mez	X			Herb	DCZ 4034
** Burmanniaceae **					
*Burmannia capitata* (Walter ex J.F.Gmel.) Mart.				Herb	MP 644
*Burmannia flava* Mart.				Herb	DCZ 3903
** Cabombaceae **					
*Cabomba furcata* Schult. & Schult.f.				Herb	DCZ 3963
** Commelinaceae **					
*Commelina erecta* L.				Herb	DCZ 4058
*Dichorisandra hexandra* (Aubl.) C.B. Clarke				Liana	DCZ 3858
** Connaraceae **					
*Rourea ligulata* Baker				Shrub	COA 666
** Convolvulaceae **					
*Distimake macrocalyx* (Ruiz & Pav.) A.R. Simões & Staples	X			Liana	MP 660
*Ipomoea decora* Meisn.				Liana	DCZ 4057
*Ipomoea marabaensis* D.F.Austin & Secco				Liana	DCZ 3873
*Ipomoea rubens* Choisy	X			Liana	MP 672
** Cucurbitaceae **					
*Gurania sinuata* (Benth.) Cogn.				Herb	AHS 2167
** Cyperaceae **					
*Bulbostylis conifera* (Kunth) C.B. Clarke				Herb	COA 624
*Cyperus aggregatus* (Willd.) Endl.				Herb	DCZ 3865
*Cyperus laxus* Lam.				Herb	DCZ 3957
*Cyperus sesquiflorus* (Torr.) Mattf. & Kük.				Herb	DCZ 4031
*Cyperus sphacelatus* Rottb.				Herb	DCZ 4042
*Diplasia karatifolia* Rich. in Pers.	X			Herb	DCZ 4032
*Eleocharis flavescens* (Poir.) Urb.				Herb	MP 627
*Eleocharis pedrovianae* C.S. Nunes, R. Trevis. & A. Gil		X		Herb	DCZ 4027
*Eleocharis plicarhachis* (Griseb.) Svenson				Herb	COA 678
*Rhynchospora barbata* (Vahl) Kunth				Herb	COA 657
*Rhynchospora filiformis* Vahl				Herb	DCZ 3930
*Rhynchospora holoschoenoides* (Rich.) Herter				Herb	MP 608
*Rhynchospora seccoi* C.S.Nunes, P.J.S. Silva Filho & A.Gil				Herb	DCZ 3905
*Scleria cyperina* Willd. ex Kunth				Herb	DCZ 3925
*Scleria microcarpa* Nees ex Kunth				Herb	COA 650
** Dioscoreaceae **					
*Dioscorea piperifolia* Humb. & Bonpl. ex Willd.				Liana	DCZ 3884
*Dioscorea trilinguis* Griseb.	X			Liana	DCZ 3934
** Eriocaulaceae **					
*Eriocaulon carajense* Moldenke		X		Herb	DCZ 3936
*Eriocaulon cinereum* R.Br.				Herb	DCZ 4049
*Paepalanthus fasciculoides* Hensold				Herb	DCZ 3878
*Syngonanthus discretifolius* (Moldenke) M.T.C. Watanabe		X		Herb	PLV 6119
*Syngonanthus heteropeplus* (Körn.) Ruhland				Herb	MP 659
** Erythroxylaceae **					
*Erythroxylum nelson-rosae* Plowman		X		Shrub	COA 672
*Erythroxylum rufum* Cav.				Shrub	COA 637
** Euphorbiaceae **					
*Alchornea discolor* Poeppig				Shrub	DCZ 3886
*Aparisthmium cordatum* (A. Juss.) Baill.				Tree	DCZ 3997
*Astraea lobata* (L.) Klotzsch				Shrub	DCZ 3955
*Mabea angustifolia* Spruce ex Benth.				Shrub	DCZ 3987
*Manihot quinquepartita* Huber ex D.J.Rogers				Shrub	DCZ 3954
*Manihot tristis* Müll.Arg.				Shrub	MP 666
*Maprounea brasiliensis* A.St.-Hil.	X			Shrub	DCZ 3991
** Fabaceae **					
*Abrus melanospermus* Hassk.				Liana	DCZ 3912
Aeschynomene sensistiva var. hispidula (Kunth) Rudd				Subshrub	DCZ 4024
*Bauhinia pulchella* Benth.				Shrub	DCZ 3869
*Camptosema ellipticum* (Desv.) Burkart				Shrub	DCZ 3907
*Centrosema carajasense* Cavalcante				Herb/Liana	DCZ 4007
*Chamaecrista desvauxii* (Collad.) Killip				Subshrub	DCZ 3946
*Clitoria falcata* Lam.				Liana	DCZ 3917
*Crotalaria maypurensis* Kunth				Shrub	DCZ 3881
*Dioclea apurensis* Kunth				Liana	DCZ 3919
*Inga calantha* Ducke	X			Tree	COA 600
*Inga heterophylla* Willd	X			Tree	DCZ 4036
*Inga leiocalycina* Benth.	X			Tree	MP 598
*Mimosa dasilvae* A.S.L. Silva & Secco	X	X	X	Subshrub	COA 622
Mimosa guilandinae var. spruceana (Benth.) Barneby				Shrub	COA 668
Mimosa skinneri Benth. var. carajarum Barneby		X		Herb	DCZ 3860
*Mimosa somnians* Humb. & Bonpl. ex Willd.				Subshrub	DCZ 3876
*Mimosa xanthocentra* Mart.				Tree	PLV 6158
*Parkia platycephala* Benth.				Shrub	DCZ 4013
*Periandra mediterranea* (Vell.) Taub.				Shrub	DCZ 3902
*Senegalia multipinnata* (Ducke) Seigler & Ebinger				Treelet	COA 603
*Stylosanthes capitata* Vogel				Subshrub	DCZ 3977
*Tachigali vulgaris* L.F.G.Silva & H.C.Lima				Tree	COA 655
** Gentianaceae **					
*Schultesia benthamiana* Klotzsch ex Griseb.				Herb	DCZ 3928
** Heliconiaceae **					
*Heliconia psittacorum* L.f.	X			Herb	MP 671
** Hypericaceae **					
*Vismia gracilis* Hieron				Treelet	COA 654
** Iridaceae **					
*Cipura xanthomela*s Maxim. ex Klatt				Herb	DCZ 3899
** Lamiaceae **					
*Amasonia lasiocaulo*s Mart. & Schau ex Schau.				Subshrub	DCZ 3947
*Hyptis atrorubens* Poit.				Herb	DCZ 3981
*Mesosphaerum pectinatum* (L.) Kuntze				Herb	MN 697
*Mesosphaerum suaveolens* (L.) Kuntze				Herb	DCZ 4048
*Vitex panshiniana* Moldenke	X			Tree	DCZ 4053
** Lauraceae **					
*Cassytha filiformis* L.				Parasite	DCZ 3874
Dicypellium aff. caryophyllaceum (Mart.) Nees	X		X	Shrub	PLV 6100
*Mezilaurus itauba* (Meisn.) Taub. ex Mez				Shrub	DCZ 4001
*Rhodostemonodaphne praeclara* (Sandwith) Madriñán	X			Tree	DCZ 3983
** Lentibulariaceae **					
*Utricularia neottioides* A.St-Hil & Girard				Herb	MP 664
*Utricularia pusilla* Vahl				Herb	DCZ 3904
*Utricularia subulata* L.				Herb	PLV 6139
** Loranthaceae **					
*Passovia pedunculata* (Jacq.) Kuijt				Parasite	DCZ 3909
*Psittacanthus eucalyptifolius* (Kunth) G. Don				Parasite	DCZ 4056
** Lythraceae **					
*Cuphea annulata* Koehne				Subshrub	DCZ 3864
*Cuphea carajasensi*s Lourteig		X		Shrub	COA 616
** Malpighiaceae **					
*Banisteriopsis malifolia* (Nees & Mart.) B.Gates				Shrub	MN 743
*Banisteriopsis stellaris* (Griseb.) B.Gates				Liana	DCZ 3863
*Byrsonima chrysophylla* Kunth				Shrub	DCZ 3929
*Heteropterys nervosa* A.Juss.				Liana	COA 645
** Malvaceae **					
*Waltheria indica* L.	X			Shrub	DCZ 4064
** Marantaceae **					
*Monotagma plurispicatum* (Körn.) K.Schum.				Herb	DCZ 4000
** Marcgraviaceae **					
*Norantea guianensis* Aubl.				Shrub	DCZ 3887
** Melastomataceae **					
*Bellucia grossularioides* (L.) Triana	X			Shrub	DCZ 3995
*Brasilianthus carajensis* Almeda & Michelangeli				Herb	DCZ 3877
*Clidemia capitellata* (Bonpl.) D.Don				Shrub	DCZ 4020
*Miconia alternans* Naudin				Shrub	DCZ 4021
*Miconia heliotropoides* Triana				Shrub	DCZ 4008
*Nepsera aquatica* (Aubl.) Naudin				Herb	COA 649
*Pleroma carajasense* K.Rocha, R.Goldenb. & F.S.Mey		X		Shrub	DCZ 3910
*Pterolepis trichotoma* (Rottb.) Cogn.				Herb	DCZ 4019
*Tibouchina edmundo*i Brade				Shrub	DCZ 3932
** Menispermaceae **					
*Abuta grandifolia* (Mart.) Sandwith				Shrub	COA 646
*Cissampelos andromorpha* DC. .				Liana	COA 663
** Metteniusaceae **					
*Emmotum nitens* (Benth.) Miers				Shrub	MP 601
** Myrtaceae **					
*Eugenia punicifolia* (Kunth) DC.				Shrub	DCZ 3894
*Myrcia cuprea* (O.Berg.) Kiaersk.				Shrub	COA 639
*Myrcia splendens* (Sw.) DC.				Shrub	DCZ 3965
*Myrciaria floribunda* (H.West ex Willd.) O.Berg				Shrub	DCZ 3915
*Myrciaria glomerata* O.Berg	X			Shrub	DCZ 4010
** Ochnaceae **					
*Ouratea castaneifolia* (DC.) Engl.				Treelet	DCZ 3920
*Ouratea cearensis* (Tiegh.) Sastre & Offroy	X			Shrub	COA 604
*Ouratea racemiformis* Ule				Shrub	DCZ 4033
** Onagraceae **					
Ludwigia cf. latifolia (Benth.) H.Hara	X			Subshrub	DCZ 3967
*Ludwigia nervosa* (Poir.) H.Hara				Shrub	COA 674
** Orchidaceae **					
*Catasetum boyi* Mansf.	X			Herb	JBFS 648
*Catasetum discolor* (Lindl.) Lindl.				Herb	DCZ 4030
*Cyrtopodium andersonii* (Lamb. ex Andrews) R.Br.				Herb	COA 643
*Encyclia chloroleuca* (Hook.) Neum.	X			Herb	JBFS 540
*Epidendrum strobiliferum* Rchb.f.	X			Herb	COA 667
*Erycina pusilla* (L.) N.H.Williams & M.W.Chase				Herb	JBFS 498
*Habenaria nuda* Lindl				Herb	MP 609
*Habenaria orchiocalcar* Hoehne	X			Herb	JBFS 219
*Polystachya concreta* (Jacq.) Garay & H.R.Sweet				Herb	COA 669
*Rodriguezia lanceolata* Ruiz & Pav.	X			Herb	COA 665
Scaphyglottis cf. livida				Herb	COA 671
*Sobralia liliastrum* Salzm. ex Lindl.				Herb	DCZ 3888
** Orobanchaceae **					
*Buchnera carajasensis* Scatigna & N.Mota		X		Herb	DCZ 3931
** Passifloraceae **					
*Passiflora ceratocarpa* F. Silveira				Liana	DCZ 4060
*Passiflora picturata* Ker Gawl.	X			Liana	DCZ 3976
*Passiflora tholozanii* Sacco				Liana	COA 612
** Phyllanthaceae **					
*Phyllanthus hyssopifolioides* Kunth.				Herb	DCZ 4028
*Phyllanthus minutulus* Müll.Arg.				Herb	DCZ 4026
** Phytolaccaceae **					
*Phytolacca thyrsiflora* Fenzl ex J. Schmidt				Herb	DCZ 4041
** Piperaceae **					
*Peperomia albopilosa* D. Monteiro		X		Herb	PLV 6169
*Peperomia magnoliifolia* (Jacq.) A.Dietr.				Herb	COA 647
** Plantaginaceae **					
*Scoparia dulcis* L.				Herb	DCZ 4065
** Poaceae **					
*Acroceras zizanioides* (Kunth) Dandy				Herb	DCZ 4022
*Andropogon bicornis* L.				Herb	DCZ 3950
Axonopus cf. longispicus (Döll) Kuhlm.				Herb	DCZ 4023
*Axonopus rupestris* Davidse				Herb	DCZ 3896
*Eleusine indica* (L.) Gaertn.*				Herb	DCZ 4045
*Hildaea parvispiculata* C. Silva & R.P. Oliveira				Herb	PLV 6124
*Ichnanthus calvescens* (Nees ex Trin.) Döll				Herb	DCZ 4011
*Luziola peruviana* Juss. ex J.F.Gmel.				Herb	DCZ 3918
*Melinis minutiflora* P.Beauv.*				Herb	COA 640
*Mesosetum cayennense* Steud.				Herb	PLV 6117
*Oryza glumaepatula* Steud.				Herb	BFF 634
*Paspalum axillare* Swallen				Herb	PLV 6130
*Paspalum foliiforme* S.Denham				Herb	DCZ 3916
*Paspalum reticulinerve* Renvoize				Herb	PLV 6166
*Rhytachne gonzalezii* Davidse				Herb	PLV 6127
*Rugoloa pilosa* (Sw.) Zuloaga				Herb	DCZ 3964
*Steinchisma laxum* (Sw.) Zuloaga				Herb	COA 677
*Taquara micrantha* (Kunth) I.L.C.Oliveira & R.P.Oliveira				Herb	DCZ 3999
*Trachypogon spicatus* (L.f.) Kuntze				Herb	DCZ 3944
Trichanthecium cf. arctum (Swallen) Zuloaga & Morrone				Herb	DCZ 3913
*Urochloa maxima* (Jacq.) R.D. Webster*				Herb	DCZ 3951
** Polygalaceae **					
*Bredemeyera divaricata* (DC.) J.F.B. Pastore				Shrub	DCZ 3911
*Caamembeca spectabilis* (DC.) J.F.B. Pastore				Subshrub	COA 642
*Polygala adenophora* DC.				Herb	DCZ 3900
** Portulacaceae **					
*Portulaca sedifolia* N.E.Br.				Herb	DCZ 3862
** Primulaceae **					
*Cybianthus detergens* Mart.				Shrub	DCZ 4062
** Proteaceae **					
*Roupala montana* Aubl.				Shrub	DCZ 4063
** Rhamnaceae **					
*Gouania pyrifolia* Reissek	X			Liana	DCZ 3953
** Rubiaceae **					
*Alibertia edulis* (Rich.) A. Rich. ex DC.				Shrub	DCZ 4035
*Borreria alata* (Aubl.) DC.				Herb	DCZ 3866
*Borreria carajasensis* E.L. Cabral & L.M. Miguel		X		Subshrub	DCZ 3859
*Borreria semiamplexicaulis* E.L.Cabral				Herb	DCZ 3938
*Cordiera myrciifolia* (K.Schum.) C.H.Perss. & Delprete				Shrub	DCZ 3971
*Coutarea hexandra* (Jacq.) K.Schum.	X			Shrub	COA 610
*Guettarda argentea* Lam.				Shrub	COA 602
*Palicourea guianensis* Aubl.				Treelet	DCZ 4052
*Perama carajensis* J.H. Kirkbr.		X		Herb	DCZ 3879
*Psychotria colorata* (Willd. ex Schult.) Mull. Arg.				Herb	DCZ 4017
*Psychotria hoffmannseggiana* (Willd. ex Schult.) Mull. Arg.				Subshrub	COA 601
*Sabicea grisea* Cham. & Schltdl.				Liana	DCZ 3901
** Rutaceae **					
*Dictyoloma vandellianum* A. Juss.				Treelet	DCZ 3975
*Ertela trifolia* (L.) Kuntze				Subshrub	COA 607
*Pilocarpus microphyllus* Stapf ex Wardlew.				Shrub	COA 653
** Salicaceae **					
*Casearia arborea* (Rich.) Urb.				Tree	DCZ 3982
*Casearia javitensis* Kunth				Shrub	DCZ 4014
** Sapindaceae **					
*Allophylus semidentatus* (Miq.) Radlk.	X			Shrub	DCZ 3959
*Paullinia stellata* Radlk.	X			Liana	DCZ 4044
*Pseudima frutescens* (Aubl.) Radlk.	X			Shrub	PLV 6151
*Serjania lethalis* A.St.-Hil.				Liana	DCZ 3996
** Sapotaceae **					
*Pouteria ramiflora* (Mart.) Radlk.				Treelet	DCZ 3969
** Simaroubaceae **					
*Simaba guianensis* Aubl.				Shrub	DCZ 3984
*Simarouba amara* Aubl.				Shrub	DCZ 3985
** Siparunaceae **					
*Siparuna ficoides* S.S.Rener & Hausner				Treelet	COA 660
** Smilacaceae **					
*Smilax irrorata* Mart. ex Griseb				Liana	DCZ 3935
** Solanaceae **					
*Solanum americanum* Mill.				Herb	DCZ 4059
*Solanum crinitum* Lam.				Treelet	COA 623
** Trigoniaceae **					
*Trigonia nivea* Cambess.				Liana	COA 651
** Turneraceae **					
*Turnera glaziovii* Urb				Shrub	DCZ 4012
*Turnera laciniata* Arbo				Herb	DCZ 3993
*Turnera melochioides* Cambess.				Shrub	PLV 6160
** Urticaceae **					
*Cecropia palmata* Willd.				Tree	COA 664
** Velloziaceae **					
*Vellozia glauca* Pohl				Herb	DCZ 3890
** Verbenaceae **					
*Lantana trifolia* L.	X			Shrub	MN 755
*Lippia grata* Schauer				Shrub	DCZ 3871
*Stachytarpheta cayennensis* (Rich.) Vahl				Subshrub	COA 608
** Vitaceae **					
*Cissus erosa* Rich.				Liana	DCZ 3882
** Vochysiaceae **					
*Qualea parviflora* Mart.				Tree	MP 624
** Xyridaceae **					
*Xyris brachysepala* Kral		X		Herb	PLV 6125
**SPECIES TOTAL (254)**	**36**	**17**	**2**		

Among the 38 edaphic endemic species of canga, defined according to Giulietti et al. (2019), 17 (c. 50%) were recorded in SFX. Two of these, *Erythroxylum nelson-rosae* Plowman (Erythroxylaceae) and *Matelea microphylla* Morillo (Apocynaceae) were not previously recorded for SFX in the list of endemic edaphic species of the canga of Carajás (Giulietti et al. 2019). One species, *Mimosa dasilvae* (Fabaceae), is only known to occur in SFX.

**Table 2.** Areas compared by this study, respective area codes used in the multivariate analysis and number of angiosperms species recorded for each area. Serra de Campos of São Félix do Xingu (SFX) data is produced by this study, ARQ-CAN data is available in Fonseca-da-Silva et al. (2020) and Flora of the canga of the Serra de Carajás (FCC) data is available in Mota et al. (2018).

Area code	Area	Species	Cumulative species
ARQ	Serra Arqueada	149	149
S11A	Serra dos Carajás – Serra Sul 11A	230	535
S11B	Serra dos Carajás – Serra Sul 11B	201	
S11C	Serra dos Carajás – Serra Sul 11C	180	
S11D	Serra dos Carajás – Serra Sul 11D	428	
SN1	Serra dos Carajás – Serra Norte 1	383	643
SN2	Serra dos Carajás – Serra Norte 2	125	
SN3	Serra dos Carajás – Serra Norte 3	218	
SN4	Serra dos Carajás – Serra Norte 4	308	
SN5	Serra dos Carajás – Serra Norte 5	293	
SN6	Serra dos Carajás – Serra Norte 6	99	
SN7	Serra dos Carajás – Serra Norte 7	112	
SN8	Serra dos Carajás – Serra Norte 8	101	
SB	Serra dos Carajás – Serra da Bocaina	223	336
ST	Serra dos Carajás – Serra do Tarzan	211	
SFX	Serra de Campos – São Félix do Xingu	248	248

Around 25% (60) of the 248 angiosperms registered for SFX are restricted to the Amazonian Rainforest biome, but the majority of the flora is widely distributed in open habitats throughout South America.

### The vegetation of the Serra de Campos

Regarding the phytophysiognomies listed by Mota et al. (2015) for the region, the canga vegetation of the SFX has a predominance of vast spreads of scrub composed of closely disposed treelets and shrubs. Amongst them, treelets and shrubs such as *Byrsonima chrysophylla* Kunth, *Cordiera myrciifolia* (K.Schum.) C.H.Perss. & Delprete, *Anemopaegma carajasense* A.H. Gentry ex Firetti-Leggieri & L.G. Lohmann*, *Cuphea annulata* Koehne, *Lippia grata* Schauer, *Erythroxylum nelson-rosae* Plowman*, *Syagrus cocoides* Mart., as well as several species of *Myrcia* and *Eugenia*, the palm *Syagrus cocoides* Mart. and scramblers and climbers such as *Norantea guianensis* Aubl., *Cissus erosa* Rich., *Mandevilla scabra* (Hoffmanns. ex Roem. & Schult.) K. Schum. and *Smilax irrorata* Mart. ex Griseb. On more exposed, bare canga slabs, the plants grow mostly in rock crevices with presence of monocots such as *Vellozia glauca* Pohl, *Sobralia liliastrum* Salzm. ex Lindl., *Dyckia duckei* L.B. Sm. and the tuberous, low growing *Mandevilla tenuifolia* (J.C. Mikan) Woodson, as well as the herbaceous *Borreria semiamplexicaulis* E.L.Cabral, *Perama carajensis* J.H.Kirk.*, *Begonia humilis* Dryand and *Brasilianthus carajensis* Almeda & Michelangeli*. The nodular canga has more or less continuous covering of grass and sedge, with occasional specimens of *Riencourtia pedunculosa* (Rich.) Prusky. During the expeditions we did not come across low forest groves, and our impression was that between the canga edge and the surrounding rainforest there was not much transition but a sharp substitution of the open vegetation by the associated forest types. Regarding the hydromorphic vegetation found in SFX, temporary shallow ponds with *Utricularia* species, *Burmannia flava* Mart., *Cabomba furcata* Schult. & Schult. f., *Syngonanthus caulescens* (Poir.) Ruhland and *Xyris brachysepala* Kral.* were visited. However, perennial, larger ponds of the magnitude found in the *Serra Sul* were lacking and temporary streams were not observed. There were also Palm swamps (*buritizais*), with margins occupied by *Mauritia flexuosa* Mart. and *Mauritiella armata* (Mart.) Burret, harbouring aquatic *Oryza glumaepatula* Steud., *Helanthium tenellum* (Mart. ex Schult. & Schult.f.) Britton and *Eleocharis* spp. (edaphic endemic species marked with *).

### Database of the flora of Serra dos Carajás complex

The biogeographical database from the CRC of the Carajás complex was updated by our study (see supplementary data) and includes now a total of 893 angiosperms distributed in 121 families and 441 genera. For the Carajás flora (FCC), Poaceae was the most species-rich family (75 species in the FCC), followed by Fabaceae (66 spp.), Cyperaceae (57 spp.), Rubiaceae (49 spp.), and Melastomataceae (40 spp.). The richest genera were *Rhynchospora* (24 spp.), *Miconia* (18 spp.), *Paspalum* and *Solanum* (17 spp. each), *Myrcia* and *Ipomoea* (13 spp. each), while 64% (284 genera) were represented by only a single species. The inclusion of SFX in our database increased the number of known taxa by 18 genera and 37 species not previously recorded for the canga of Carajás.

### Biogeography of the Campos Rupestres on Canga of the Carajás complex

**Figure 3.**
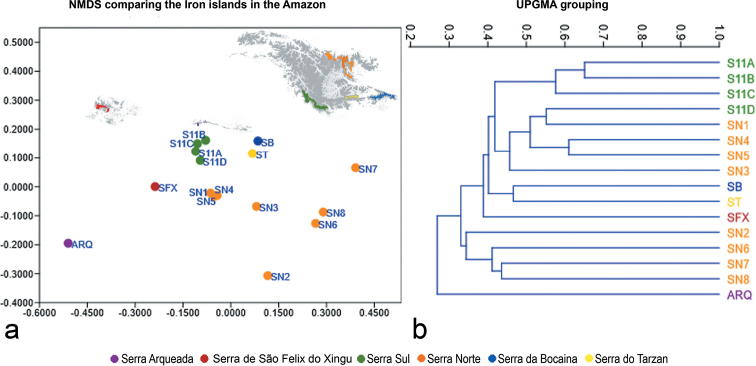
UPGMA (**a**) and NMDS (**b**) multivariate analysis clustering areas from FCC and SFX (see Table 2 for area codes). UPGMA cophenetic coefficient: 0.902. b. NMDS stress: 0.1859.

The mean angiosperm species richness for each outcrop of the Serra dos Carajás was 218 species. The NMDS and UPGMA analyses included 3451 records of 893 species across 16 sites (Fig. 3a, b). The UPGMA analyses produced statistically significant clusters (Fig. 3b) with the same major groups found by Fonseca-da-Silva et al. (2020), one comprising four of the eight areas of the Serra Norte (SN2, SN6, SN7, and SN8), while the remaining four (SN1, SN3, SN4, and N5) appear closer to the areas of Serra Sul (S11A, S11B. S11C, S11D), along with SB and ST. SA also emerged as the least similar to the Carajás complex, and SFX was found to be more similar to the group comprising SB, ST, Serra Sul and the four most species rich sites in Serra Norte (SN1, SN3, SN4, and SN5). A similar result was obtained by the NMDS analysis (Fig. 3a), also showing SA as the most dissimilar from other areas.

**Figure 4.**
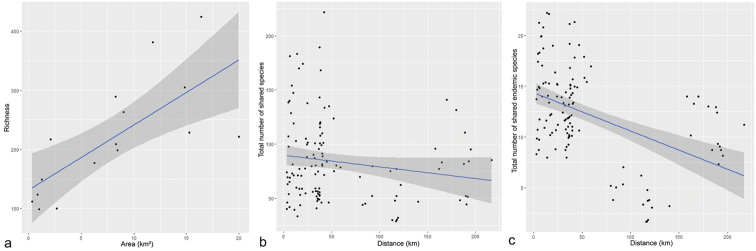
**a** Species richness plotted against area of Carajás. Pearson correlation coefficients: *r* = 0.806094, *P* = 0.001548 **b** the number of species shared between site pairs does not change significantly with geographical distance between regions. *r* = -0.16; *P* = 0.08 **c** the number of shared endemic species between site pairs declines with geographical distance between regions. *r*= -0.45872; *P* = 1.37e-07.

Species richness was significantly correlated with site area (*r* = 0.806094, *P* = 0.001548). The larger the area of each individual mountaintop (site), the larger the number of species recorded. The total number of shared species between mountaintop outcrops did not differ significantly with geographical distance across sites (*r* = -0.16; *P* = 0.08). There was a tendency of distant sites to share less species, but this trend was not significant. When the residuals of this model were evaluated they significantly departed from normality. Spearman’s correlation was not significant either (*p-value* = 0.2972). However, when focusing on the number of shared endemic edaphic species versus the geographical distance between sites, we found a significant correlation, where closer sites shared more edaphic endemic species than with more distant sites (*r* = -0.45872; *P* = 1.37e-07) (Fig. 4).

Regarding the total of species of the canga, the Carajás iron islands share an average of 40% of their flora with each other. SFX has, on average, 30% of shared species with each other area. The percentage of similarity between sites was a minimum of 30% and a maximum of 55%.

## Discussion

### Floristic composition of Serra de Campos × other canga outcrops

The most species-rich families and genera found in the SFX coincide with those found in the Flora das cangas de Carajás (Mota et al. 2018) and SA (Fonseca-da-Silva et al. 2020), where Cyperaceae, Fabaceae, Poaceae, and Rubiaceae are among the richest plant families. Interestingly, SFX has a much higher number of Orchidaceae species than other surveys of canga in the Amazon (Koch et al. 2018; Mota et al. 2018; Fonseca-da-Silva et al. 2020). The participation of botanical specialists during collecting expeditions enhances floristic studies in the Amazon (Medeiros et al. 2014) and elsewhere, and the high number of Orchidaceae in SFX possibly reflects the specific search for this group by J.B. Silva in the region from the 1990’s onwards, which may have resulted in a greater sampling effort for this group when compared to other areas.

There is a large turnover of species between outcrops (Zappi et al. 2019; Fonseca-da-Silva et al. 2020) and very few species are widely distributed across these disjunct, isolated habitats. Similar to what was found by (Costa et al. 2019) in Amazonian White Sand Campinas, the isolation of the patchy canga outcrops limits dispersal and increases floristic differentiation, and the adverse conditions, such as high temperature, extreme exposure to sunlight and winds, and a relatively well defined dry season represent ecological filters for the species that occupy the canga, partly explaining the high number of endemic species in the CRC of Carajás.

As an example, only three species were recorded in all surveyed areas: the widely distributed *Riencourtia pedunculosa*, an Asteraceae common in open areas in the Amazon (Flora do Brasil under construction, Bringel 2014), and two species associated with Amazonian canga outcrops: *Brasilianthus carajensis* and *Perama carajensis*. *Perama carajensis* is a confirmed canga edaphic endemic species, and *Brasilianthus carajensis* has been collected also on granite, being locally endemic to Carajás, but not a canga edaphic endemic (Giulietti et al. 2019; Silva et al. 2020). Other four species also present wide occurrence across *campos rupestres* on canga of Carajás: *Bulbostylis conifera* (Kunth) C.B. Clarke, *Rhynchospora barbata* (Vahl) Kunth, *Rhynchospora seccoi* C.S.Nunes et al., and *Syngonanthus discretifolius* (Moldenke) M.T.C. Watanabe were recorded for SFX and many other FCC areas, except for one of them missing in SN3, SN7, SN7 and SA, respectively. Their absence in these four sites may be related to the more modest canga surface found in these areas.

Some widely distributed species from the canga of Carajás, found at more than 10 of the 16 sites surveyed, were not recorded at SFX. The absence of the common treelets *Callisthene microphylla* Warm. and Mimosa acutistipula var. ferrea Barneby (Mota et al. 2015) at SFX may be partially explained by differences in the micro-habitats between SFX and the other canga outcrops considered here. For *Brasilianthus carajensis*, distinct adaptive genetic clusters have been found in the SFX (see Silva et al. 2020), increasing the argument for the protection of the site.

The canga is typically a mosaic of different vegetation types (Mota et al. 2015, Viana et al. 2016). Some of these vegetation types are infrequent in SFX, as for example low forest groves (Mota et al. 2015), and in consequence some of the species found in these groves elsewhere are absent at SFX: *Callisthene microphylla*, Mimosa acutistipula var. ferrea, and *Cereus hexagonus* (L.) Mill. Although forest groves are closely associated with the lower scrub vegetation, the latter is more abundant in the canga plateau of SFX than the former. In plateau SFX2 of SFX the shrubby vegetation is dominant, and there are large stands of *Syagrus cocoides* Mart., a palm emerging from the impenetrable shrubbery. In the context of CRC of Carajás, this palm forms large populations only in SA and SFX.

Despite having the lowest number of species registered in the FCC, the hydromorphic vegetation found atop the plateaus is the habitat with the highest proportion of exclusive species (Pereira et al. 2016; Mota et al. 2018). Seasonal lakes and palm lakes in the SFX ensure the presence of annual aquatic species such as *Eriocaulon carajense* Moldenke, *Oryza glumaepatula* Steud., *Syngonanthus caulescens* (Poir.) Ruhland, and *Xyris brachysepala* Kral.

As a relatively large canga site isolated from the active iron mines further to the east, the SFX has been found to harbour a rich and unique vegetation, representing a suitable area for the implementation of conservation strategies. On the other hand, this canga outcrop is currently threatened by surrounding deforestation, land transformation and frequent fires, and is not included within any type of protected area.

### Iron islands of Carajás and their floristic connections

The mosaic of landscapes typical of CRC of Carajás may also explain the low floristic similarity between the sites. The number of shared species represents less than half the local richness from each site separately. This brings attention to the high beta diversity among sites (Zappi et al. 2019), with a large species turnover across these disjunct outcrops. Habitat diversity associated with the size of the island-like habitats is also related to the beta diversity in French Guiana´s inselbergs (Henneron et al. 2019), similarly to what is found in Andean alpine flora (Sklenář et al. 2014) and South American tepuis (Riina et al. 2019). This confirms the association between area and habitat diversity found here for the canga vegetation as an important factor for determining plant biodiversity.

The greater similarity between SFX, SB and ST, along with *Serra Sul* (S11A, S11B, S11C, and S11D) and SN1, SN3, SN4 and SN5 reflected in the UPGMA clustering patterns (Fig. 3b) suggests there is more similarity of species richness between the largest sites rather than among geographically closest areas, as observed by Fonseca-da-Silva et al. (2020) for SA. In fact, the correlation between the shared species of each canga site and their geographical distance was significant. Considering the size of each of these areas and their positive correlation with floristic richness (Fig. 4), we interpret the canga’s overall surface as being more important for floristic composition than the distance between sites in the Serra dos Carajás. Thus, the larger a canga outcrop is, the greater the number of micro-habitats it can harbour, reflecting an increased species richness and unique floristic composition of each canga site. On the other hand, that relationship (distance between areas vs shared flora) holds true when analysing shared endemic species, where shared endemic species decrease with distance at different rates (Fig. 4C).

The low number of species restricted to the Amazon (25%) and the high number of species widely distributed in South America (75%) recorded at SFX, may explain the discrepancy in the correlation between shared species and distance being negative when all species are considered, whereas it is positive for endemic species only. On a macro-scale, the majority of the species recorded in SFX have a broad distribution, occurring beyond the Amazon Rainforest, and the distance factor between different outcrops may not matter so much. On the other hand, when observing only the species endemic to Carajás, and especially edaphic endemic species, the trend is the opposite, possibly due to the local scale of observation, as elsewhere the distance between areas tends to affect the floristic similarity between island vegetations (Sklenář et al. 2014; Schrader et al. 2020). A genomic study revealed that gene flow in two endemic species of Carajás is mainly influenced by geographic distance between mountain pairs, as the rainforest surrounding different mountaintops constitutes an important barrier (Carvalho et al. 2019). Therefore, gene flow also decreases with the increase of the barrier represented by the rainforest (Carvalho et al. 2019).

Another factor that may have an impact on the contrasting effects of floristic similarity vs. distance from canga islands is the different environmental requirements of herbs, shrubs and trees, that shape their biogeographical patterns and affect species-area and richness-environment relationships (Schrader et al. 2020). Herbs, shrubs and trees have contrasting strategies in different environmental conditions with potential implications for community assemblage on islands. For example, herbs can form larger populations on small islands due to their smaller size, and as a result face less risk of extinction and greater dispersal capacity (Moles 2005; Thomson et al. 2010), while shrubs are associated with more stable environmental conditions, and therefore have more success on larger islands (Chiarucci et al. 2017).

Recent analyses of open vegetation in the Amazon reinforce the insular character of Amazonian canga and their low similarity to other vegetation types in the Amazonian biome (Devecchi et al. 2020). While there is some evidence that canga in Southeastern Brazil may be influenced by the surrounding Atlantic Rainforest and Cerrado (Zappi et al. 2017) these biomes are known to have a more varied life-form balance (respectively 1: 4 and 1: 7 proportion of trees over other life forms) than the Amazon Rainforest, where the life form balance is less extreme (1: 2) (Brazil Flora Group [BFG] 2015), thus it may have less floristic influence over the open vegetation found in the CRC of Carajás (Zappi et al. 2019). Therefore, in order to colonize the Amazonian CRC, shrubby or herbaceous plant species may have to come from further afield through long distance dispersal, and, if established, they may remain genetically isolated from their original populations, leading over a period of time to the patterns of endemism observed today.

**Table 3.** Species richness of the iron islands outcrops of Carajás complex (bold diagonal) along with the number of shared species (above diagonal) and distance in kilometres (below diagonal) between the centroid sites; an estimated area for each site is provided.

Sites	Area (km^2^)	SB	ST	ARQ	S11A	S11B	S11C	S11D	SFX	SN1	SN2	SN3	SN4	SN5	SN6	SN7	SN8
SB	19.98	**221**	100	47	79	80	75	135	85	124	46	84	108	101	56	57	56
ST	8.3	24	**209**	48	88	90	80	138	84	119	59	87	102	105	55	59	53
ARQ	1.27	140	116	**149**	52	44	45	80	70	75	30	52	77	62	30	29	32
S11A	15.27	59	24	92	**228**	139	119	170	96	143	59	89	116	101	56	54	53
S11B	8.44	54.6	30.8	82	4.5	**199**	107	147	77	120	53	81	96	99	49	52	48
S11C	6.26	52.5	28.8	85	10	4.5	**177**	140	83	110	46	72	101	91	49	41	50
S11D	16.41	47	24.4	92.3	15.7	9.8	5.7	**424**	141	222	80	134	189	168	75	80	72
SFX	9.04	217	193	79.5	158	162	165	170	**239**	131	48	82	111	95	52	44	51
SN1	11.81	52	37.7	111	37	38	40	42	180	**381**	98	154	183	174	77	71	78
SN2	0.86	46.8	32.8	113	36.8	37.1	39.3	40	184	5.18	**124**	69	73	71	40	34	44
SN3	2.1	44.7	32	117.5	40.2	40.1	42	42.2	188	8.1	3.8	**217**	129	103	71	60	59
SN4	14.83	38	25	117.4	37.5	36.4	37.7	37	189	13.7	8.6	7.4	**305**	181	74	65	81
SN5	8.26	32.36	22.75	122	41	39	40	38.53	195	19.78	14.6	12.4	6.2	**289**	63	54	69
SN6	0.97	35.29	22.46	118	37.3	35.8	36.7	35.7	190	16	11	10	3	4	**99**	40	42
SN7	0.34	33	19	117	35.7	33.8	34	33.1	190.5	18	14	13	6	5	3	**112**	46
SN8	2.69	30	17	119	37	34.7	35	33	192	22	17	16	8.8	6	5.7	3.3	**100**

Different evolutionary processes of the species occurring in CRC may also have led to different floristic composition in the outcrops. Although evolutionary studies involving species of canga in the Brazilian Amazon are just beginning (Zappi et al. 2017), the phylogeography of a species of Gesneriaceae distributed in humid rock formations in the Cerrado reveals its recent expansion into CRC vegetation during the Pleistocene (Fiorini et al. 2020). Recent and rapid radiations have been observed in mountaintops ecosystems (Salerno et al. 2012; Pirie et al. 2016; Vasconcelos et al. 2020) but more phylogenetic and phylogeographic studies are necessary to establish dating for plants species groups found in the CRC in order to understand their diversification and colonization processes.

**Table 4.** Endemic edaphic species of the iron islands outcrops of Carajás complex (bold diagonal) along with the number of shared endemic species (above diagonal) and distance in kilometres (below diagonal) between the centroid sites.

Sites	SB	ST	ARQ	S11A	S11B	S11C	S11D	SFX	SN1	SN2	SN3	SN4	SN5	SN6	SN7	SN8
SB	**20**	15	3	17	15	16	19	11	18	11	15	15	13	11	11	12
ST	24	**16**	2	14	13	14	15	9	15	9	12	11	11	9	10	10
ARQ	140	116	**7**	5	4	5	7	5	6	3	4	5	3	2	2	4
S11A	59	24	92	**24**	17	21	22	14	21	10	16	17	13	11	9	12
S11B	54.6	30.8	82	4.5	**18**	18	19	10	15	14	14	13	12	10	8	10
S11C	52.5	28.8	85	10	4.5	**21**	21	13	11	10	15	15	13	10	9	12
S11D	47	24.4	92.3	15.7	9.8	5.7	**25**	14	21	11	18	19	14	12	12	14
SFX	217	193	79.5	158	162	165	170	**17**	13	9	13	12	8	9	7	9
SN1	52	37.7	111	37	38	40	42	180	**29**	15	20	22	19	13	12	16
SN2	46.8	32.8	113	36.8	37.1	39.3	40	184	5.18	**16**	15	14	14	11	8	12
SN3	44.7	32	117.5	40.2	40.1	42	42.2	188	8.1	3.8	**23**	20	15	15	12	15
SN4	38	25	117.4	37.5	36.4	37.7	37	189	13.7	8.6	7.4	**24**	18	14	12	17
SN5	32.36	22.75	122	41	39	40	38.53	195	19.78	14.6	12.4	6.2	**20**	11	9	15
SN6	35.29	22.46	118	37.3	35.8	36.7	35.7	190	16	11	10	3	4	**15**	8	10
SN7	33	19	117	35.7	33.8	34	33.1	190.5	18	14	13	6	5	3	**14**	10
SN8	30	17	119	37	34.7	35	33	192	22	17	16	8.8	6	5.7	3.3	**17**

## Conclusions

This is the most complete study analysing a database of canga outcrop islands in the Amazon thus far. Our data suggest higher shared similarity between largest sites and higher species richness. We show that species richness in these vegetation islands reveals complex biogeographic patterns and relatively high beta diversity. Outcrop size seemed to be more important than geographical proximity between outcrops, and this should be taken into account when drafting conservation and compensation measures for the canga. There are still inaccessible canga outcrops towards the north of the state of Pará that remain unexplored, and their study would certainly yield interesting information to be added to the present findings.

## Supplementary materials

Supplementary material 1 Investigating plant beta diversity of canga outcrops Data type species data https://binary.pensoft.net/file/468401This dataset is made available under the Open Database License (http://opendatacommons.org/licenses/odbl/1.0/). The Open Database License (ODbL) is a license agreement intended to allow users to freely share, modify, and use this Dataset while maintaining this same freedom for others, provided that the original source and author(s) are credited.Caroline Oliveira Andrino, Rafael Gomes Barbosa Silva, Juliana Lovo, Pedro Lage Viana, Marcelo Freire Moro, Daniela Cristina Zappi
