# Supplementary material for: Iron islands in the Amazon: investigating plant beta diversity of canga outcrops
Source: PhytoKeys. 2020 Oct 28;165:1–25. doi: 10.3897/phytokeys.165.54819 (PMC7642173; doi:10.3897/phytokeys.165.54819)
Supplement: Supplementary material 1 — Investigating plant beta diversity of canga outcrops [file phytokeys-165-001-s001.pdf]

FAMILY	GENUS	CF	SP1	AUTHOR1	RANK1	SP2	AUTHOR2	COLLECTOR	NUMBER	COLLYY	COUNTRY	MAJORAREA	MINORAREA	GAZETTEER	PLANTDESC
Acanthaceae	Justicia		birae	A.S.Reis, F.A.Silva, A.Gil & Kameyama				Pastore, M.	600	2017	Brasil	Pará	São Félix do Xingu	Serra dos Carajás	Erva ca. 40 cm alt. Folhas levemente discolores. Cálice verde; corola vermelha; anteras amarelas; estilete e filetes brancos.
Alismataceae	Limnocharis		flava	(L.) Buchenau				Viana, P.L.	6149	2016	Brasil	Pará	São Félix do Xingu	Serra de Campos	Aquática com botôes verdes
Alismataceae	Helanthium		tenellum	(Mart. ex Schult & Schult.f.) Britton				Falcão, B.F.	642	2016	Brasil	Pará	São Félix do Xingu	Serra da Seringa	Erva aquática. Corola esbranquiçada.. Pto. E1SER08JUN.
Alismataceae	Helanthium		tenellum	(Mart. ex Schult & Schult.f.) Britton				Pastore, M.	613	2017	Brasil	Pará	São Félix do Xingu	Serra dos Carajás, Serra de Campos	Erva aquática. Corola branca. ITV 1965.
Anacardiaceae	Spondias		mombin	L.				Zappi, D.C.	3921	2018	Brasil	Pará	São Félix do Xingu	Serra de Campos, 20 km N de Tancredo Neves	Arvoreta 2,5 m alt., estéril
Anacardiaceae	Anacardium		occidentale	L.				Zappi, D.C.	3923	2018	Brasil	Pará	São Félix do Xingu	Serra de Campos, 20 km N de Tancredo Neves	Arvoreta 4 m alt., estéril
Annonaceae	Xylopia		aromatica	(Lam.) Mart.				Zappi, D.C.	3970	2018	Brasil	Pará	São Félix do Xingu	Serra de Campos, 20 km N de Tancredo Neves	Arvoreta esgalhada, 3 m alt., estéril
Annonaceae	Xylopia		aromatica	(Lam.) Mart.				Nogueira, M.G.C.	730	2017	Brasil	Pará	São Félix do Xingu	Serra de Campos, estrada no interior da Serra de Campos.	Arbusto muito ramificado, desde a base, até 4,5 m de altura. Folhas discolores com face superior verde mais escuro. Cálice verde, corola com pétalas superiores verdes com margem castanho avermelhada.
Annonaceae	Annona		exsucca	DC.				Andrino, C.O.	658	2019	Brasil	Pará	São Félix do Xingu	Serra de Campos	Árvore 5 m. Frutos verdes.
Annonaceae	Guatteria		procera	R.E.Fr.				Zappi, D.C.	4050	2018	Brasil	Pará	São Félix do Xingu	Serra de Campos, 20 km N de Tancredo Neves	Árvore 8 m alt., na borda do capão, estéril
Annonaceae	Annona		sericea	Dunal				Zappi, D.C.	4051	2018	Brasil	Pará	São Félix do Xingu	Serra de Campos, 20 km N de Tancredo Neves	Arbusto esgalhado, 3 m alt., na beira do capão, botões creme
Annonaceae	Annona		sericea	Dunal				Andrino, C.O.	659	2019	Brasil	Pará	São Félix do Xingu	Serra de Campos	Árvore 5 m. Folhas discolores e frutos creme esverdeados. ITV21712
Apocynaceae	Himatanthus		articulatus	(Vahl) Woodson				Andrino, C.O.	676	2019	Brasil	Pará	São Félix do Xingu	Serra de Campos	Árvore 5 m alt, latex branco. Estéril.
Apocynaceae	Tabernaemontana		flavicans	Willd. ex Roem. & Schult.				Andrino, C.O.	613	2019	Brasil	Pará	São Félix do Xingu	Serra de Campos	Corola alva, fauce amarela.
Apocynaceae	Tabernaemontana		macrocalyx					Andrino, C.O.	605	2019	Brasil	Pará	São Félix do Xingu	Serra de Campos	Arvoreta 2 m. Cálice róseo e corola branco

Apocynaceae	Matelea	microphylla	Morillo	Zappi, D.C.	3942	2018	Brasil	Pará	São Félix do Xingu	Serra de Campos, 20 km N de Tancredo Neves	Erva trepadeira, flores verdes com coluna amarela, frutos verdes verrucosos
Apocynaceae	Mandevilla	scabra	(Hoffmanns. ex Roem. & Schult.) K. Schum.	Zappi, D.C.	3880	2018	Brasil	Pará	São Félix do Xingu	Serra de Campos, 20 km N de Tancredo Neves	Trepadeira, flores amarelas com fauce avermelhada
Apocynaceae	Mandevilla	scabra	(Hoffmanns. ex Roem. & Schult.) K. Schum.	Pastore, M.	661	2017	Brasil	Pará	São Félix do Xingu	Serra dos Carajás, Serra de Campos	Trepadeira volúvel. Folhas discolores. Cálice verde; corola amarela com a base do tubo avermelhado e interior do tubo com estrias laranja. Frutos verdes a vináceos.
Apocynaceae	Mandevilla	scabra	(Hoffmanns. ex Roem. & Schult.) K. Schum.	Nogueira, M.G.C.	721	2017	Brasil	Pará	São Félix do Xingu	Serra de Campos, estrada no interior da Serra de Campos	Trepadeira, corola amarelada com tom avermelhado, lobos internamente amarelos.
Apocynaceae	Mandevilla	tenuifolia	(J.C. Mikan) Woodson	Zappi, D.C.	3885	2018	Brasil	Pará	São Félix do Xingu	Serra de Campos, 20 km N de Tancredo Neves	Erva latescente, flores roxas com fauce creme-amarelada
Araceae	Anthurium	gracile		Zappi	5017	2019	Brasil	Pará	São Félix do Xingu	Serra de Campos	Erva epífita. Escapo, espata e espádice vináceos. ITV21714
Araceae	Anthurium	sp1		Zappi, D.C.	3898	2018	Brasil	Pará	São Félix do Xingu	Serra de Campos, 20 km N de Tancredo Neves	Erva entre rochas, inflorescência verde
Araceae	Anthurium	sp1		Zappi, D.C.	4038	2018	Brasil	Pará	São Félix do Xingu	Serra de Campos, 20 km N de Tancredo Neves	Erva com raízes suculentas, inflorescência esverdeada
Arecaceae	Mauritiella	armata	(Mart.) Burret	Zappi, D.C.	3960	2018	Brasil	Pará	São Félix do Xingu	Serra de Campos, 20 km N de Tancredo Neves	Palmeira 6 m alt., tronco espinescente, em solo alagado
Arecaceae	Syagrus	cocoides	Mart.	Zappi, D.C.	3892	2018	Brasil	Pará	São Félix do Xingu	Serra de Campos, 20 km N de Tancredo Neves	Palmeiras delgadas até 6 m alt., frutos oblongos verdes
Arecaceae	Syagrus	cocoides	Mart.	Viana, P.L.	6153	0	Brasil	Pará	São Félix do Xingu	Serra de Campos	Palmeira de estipe único, delgado (até 15 cm de diâmetro), até 4 m de altura. Planta abundande na vegetação de canga, onde molda esta fitofisionomia localmente. Folhas arqueadas, 100-125

											cm. na amostra coletada. Indivíduos em frut
Arecaceae	Syagrus	cocoides	Mart.	Falcão, B.F.	630	2016	Brasil	Pará	São Félix do Xingu	Serra da Seringa	Árvore em canga. Frutos verdes.. Pto. E1SER02JUN. DNA ITV2366
Arecaceae	Syagrus	cocoides	Mart.	Nogueira, M.G.C.	745	2017	Brasil	Pará	São Félix do Xingu	Serra de Campos	Árvore até 3 m de altura, sobre canga intemperizada, flores amarelas e frutos verdes carnosos e suculentos.
Arecaceae	Oenocarpus	distichus	Mart.	Zappi, D.C.	3948	2018	Brasil	Pará	São Félix do Xingu	Serra de Campos, 20 km N de Tancredo Neves	Palmeira 8 m alt., folhas dísticas
Asteraceae	Tilesia	baccata	(L.f.) Pruski	Zappi, D.C.	3980	2018	Brasil	Pará	São Félix do Xingu	Serra de Campos, 20 km N de Tancredo Neves	Erva 1 m alt., capítulos verdes com flores alaranjadas
Asteraceae	Unxia	camphorata	L.f.	Zappi, D.C.	3941	2018	Brasil	Pará	São Félix do Xingu	Serra de Campos, 20 km N de Tancredo Neves	Erva 20 cm alt, delicada, capítulos amarelos
Asteraceae	Unxia	camphorata	L.f.	Nogueira, M.G.C.	742	2017	Brasil	Pará	São Félix do Xingu	Serra de Campos	Erva ramificada, ca. de 60 cm de altura, cálice verde e corola amarela. Na sombra da mata.
Asteraceae	Monogereion	carajensis	G.M. Barroso & R.M. King	Zappi, D.C.	3861	2018	Brasil	Pará	São Félix do Xingu	Serra de Campos, 20 km N de Tancredo Neves	Erva com folhas viscosas, avermelhadas, capítulos rosa
Asteraceae	Monogereion	carajensis	G.M. Barroso & R.M. King	Viana, P.L.	6113	0	Brasil	Pará	São Félix do Xingu	Serra de Campos	
Asteraceae	Monogereion	carajensis	G.M. Barroso & R.M. King	Viana, P.L.	6154	0	Brasil	Pará	São Félix do Xingu	Serra de Campos	
Asteraceae	Monogereion	carajensis	G.M. Barroso & R.M. King	Pastore, M.	633	2017	Brasil	Pará	São Félix do Xingu	Serra dos Carajás	Erva de 30 cm à 1,60 m de altura. Flores com corola rosa a branca. ITV 1955.
Asteraceae	Monogereion	carajensis	G.M. Barroso & R.M. King	Nogueira, M.G.C.	700	2017	Brasil	Pará	São Félix do Xingu	Serra de Campos	Erva até 1 m altura, folhas pegajosas, invólucro verde, flores lilases.
Asteraceae	Emilia	fosbergii	Nicolson	Zappi, D.C.	4046	2018	Brasil	Pará	São Félix do Xingu	Serra de Campos, 20 km N de Tancredo Neves	Erva 30 cm alt., em campo perturbado, flores rosadas, apenas foto
Asteraceae	Riencourtia	pedunculosa	(Rich.) Pruski	Zappi, D.C.	3924	2018	Brasil	Pará	São Félix do Xingu	Serra de Campos, 20 km N de Tancredo Neves	Erva delicada, flores alvas
Asteraceae	Riencourtia	pedunculosa	(Rich.) Pruski	Viana, P.L.	6129	0	Brasil	Pará	São Félix do Xingu	Serra de Campos	
Asteraceae	Riencourtia	pedunculosa	(Rich.) Pruski	Pastore, M.	614	2017	Brasil	Pará	São Félix do Xingu	Serra dos Carajás	Erva ca. 50 cm alt. Capítulos brancos. ITV 1952.

Asteraceae	Ichthyothere	terminalis	(Spreng.) S.F. Blake	Zappi, D.C.	3868	2018	Brasil	Pará	São Félix do Xingu	Serra de Campos, 20 km N de Tancredo Neves	Arbusto 1,2 m alt., capítulos alvos com anteras arroxeadas
Asteraceae	Ichthyothere	terminalis	(Spreng.) S.F. Blake	Zappi, D.C.	3990	2018	Brasil	Pará	São Félix do Xingu	Serra de Campos, 20 km N de Tancredo Neves	Erva no subosque, 40 cm alt., capiítulos creme
Asteraceae	Ichthyothere	terminalis	(Spreng.) S.F. Blake	Viana, P.L.	6175	0	Brasil	Pará	São Félix do Xingu	Serra de Campos	
Asteraceae	Ichthyothere	terminalis	(Spreng.) S.F. Blake	Pastore, M.	667	2017	Brasil	Pará	São Félix do Xingu	Serra dos Carajás	Arbusto ca. 2 m alt. Folhas discolores. Capítulos com corola branca; anteras negras.
Begoniaceae	Begonia	humilis	Dryand	Zappi, D.C.	4029	2018	Brasil	Pará	São Félix do Xingu	Serra de Campos, 20 km N de Tancredo Neves	Erva 10-15 cm alt., caules vermelhos, flores creme
Begoniaceae	Begonia	humilis	Dryand	Viana, P.L.	6176	0	Brasil	Pará	São Félix do Xingu	Serra de Campos	
Begoniaceae	Begonia	humilis	Dryand	Pastore, M.	603	2017	Brasil	Pará	São Félix do Xingu	Serra dos Carajás	Erva delicada; ramos vermelhos. Folhas discolores. Cálice verde; corola branca; anteras creme. Frutos verdes. ITV 7163.
Bignoniaceae	Anemopaegma	carajasense	A.H. Gentry ex Firetti-Leggieri & L.G. Lohmann	Zappi, D.C.	3914	2018	Brasil	Pará	São Félix do Xingu	Serra de Campos, 20 km N de Tancredo Neves	Arbusto pouco ramificado, flores creme com fauce amarelada
Bignoniaceae	Pachyptera	incarnata	(Aubl.) Francisco & L.G. Lohmann	Zappi, D.C.	4061	2018	Brasil	Pará	São Félix do Xingu	Serra de Campos, 20 km N de Tancredo Neves	Trepadeira robusta, inflorescência esverdeada, corola creme com fauce amarela
Bignoniaceae	Anemopaegma	longipetiolatum	Sprague	Zappi, D.C.	3867	2018	Brasil	Pará	São Félix do Xingu	Serra de Campos, 20 km N de Tancredo Neves	Trepadeira com flor amarela passada, frutos verdes
Bignoniaceae	Amphilophium	mansoanum	(DC.) L.G.Lohmann	Zappi, D.C.	4025	2018	Brasil	Pará	São Félix do Xingu	Serra de Campos, 20 km N de Tancredo Neves	Trepadeira na beira da canga, flores creme
Bignoniaceae	Pleonotoma	melioides	(S.Moore) A.H.Gentry	Andrino, C.O.	638	2019	Brasil	Pará	São Félix do Xingu	Serra de Campos	Trepadeira. Flores cálice verde, corola alva com fauce creme a amarelada.
Bignoniaceae	Pleonotoma	orientalis	Sandwith	Zappi, D.C.	3883	2018	Brasil	Pará	São Félix do Xingu	Serra de Campos, 20 km N de	Trepadeira, estéril

										Tancredo Neves	
Bignoniaceae	Adenocalymma	schomburgkii	(DC.) L.G.Lohmann	Andrino, C.O.	611	2019	Brasil	Pará	São Félix do Xingu	Serra de Campos	Arbusto escandente 1,5 m. Botões verdes.
Bignoniaceae	Jacaranda	ulei	Bureau & K.Schum.	Zappi, D.C.	3945	2018	Brasil	Pará	São Félix do Xingu	Serra de Campos, 20 km N de Tancredo Neves	Arbusto monocaule, folhas buladas, estéril
Bignoniaceae	Jacaranda	ulei	Bureau & K.Schum.	Falcão, B.F.	644	2016	Brasil	Pará	São Félix do Xingu	Serra da Seringa	Arbustivo. Frutos verdes.. Pto. E1SER08JUN.
Bixaceae	Cochlospermum	orinocense	(Kunth) Steud.	Zappi, D.C.	3875	2018	Brasil	Pará	São Félix do Xingu	Serra de Campos, 20 km N de Tancredo Neves	Arvoreta 5 m alt., estéril
Bixaceae	Cochlospermum	orinocense	(Kunth) Steud.	Andrino, C.O.	614	2019	Brasil	Pará	São Félix do Xingu	Serra de Campos	Árvore 8 m. Frutos castanhos claros.
Boraginaceae	Cordia	nodosa	Lam.	Andrino, C.O.	641	2019	Brasil	Pará	São Félix do Xingu	Serra de Campos	Árvore 3 m. Inflorescência passada.
Bromeliaceae	Tillandsia	adpressiflora	Mez	Zappi, D.C.	4034	2018	Brasil	Pará	São Félix do Xingu	Serra de Campos, 20 km N de Tancredo Neves	Epífita sobre arvoretas, folhas verde-claras acinzentadas com bainha internamente atropurpúrea, inflorescência amarelo-ouro com flores azuis
Bromeliaceae	Tillandsia	adpressiflora	Mez	Viana, P.L.	6109	0	Brasil	Pará	São Félix do Xingu	Serra de Campos	
Bromeliaceae	Tillandsia	adpressiflora	Mez	Nogueira, M.G.C.	734	2017	Brasil	Pará	São Félix do Xingu	Serra de Campos	Erva epífita, cerca de 1,5 m florida, flores passadas, epífita em Melastomataceae sobre canga.
Bromeliaceae	Ananas	ananassoides	(Baker) L.B. Sm.	Zappi, D.C.	3891	2018	Brasil	Pará	São Félix do Xingu	Serra de Campos, 20 km N de Tancredo Neves	Erva em roseta, folhas avermelhadas, fruto amarelo-claro
Bromeliaceae	Ananas	ananassoides	(Baker) L.B. Sm.	Nogueira, M.G.C.	736	2017	Brasil	Pará	São Félix do Xingu	Serra de Campos	Erva terrestre com cerca de 70 cm florida, lâminas verde a cinéreas, fruto carnoso com três mudas, pendente e encostando no solo de canga, onde provavelmente ocorre a propagação.
Bromeliaceae	Aechmea	castelnavii	Baker	Andrino, C.O.	670	2019	Brasil	Pará	São Félix do Xingu	Serra de Campos	Erva rupícola. Inflorescência passadas, brácteas do escapo roseas.
Bromeliaceae	Dyckia	duckei	L.B.Sm.	Zappi, D.C.	3872	2018	Brasil	Pará	São Félix do Xingu	Serra de Campos, 20 km N de Tancredo Neves	Erva com folhas cinéreas, suculentas, flores amarelo-alaranjadas, escapos atingindo 60 cm compr., frutos enegrecidos
Bromeliaceae	Dyckia	duckei	L.B.Sm.	Nogueira, M.G.C.	735	2017	Brasil	Pará	São Félix do Xingu	Serra de Campos	Erva terrestre com cerca de 50 cm florida, lâminas argenteas a avermelhadas, flores laranja vivo com muito pólen, odor adocicado. Populações bem distribuídas em solos pedregosos sobre a canga.

Bromeliaceae	Aechmea	mertensii	(G.Mey.) Schult. & Schult.f.	Andrino, C.O.	673	2019	Brasil	Pará	São Félix do Xingu	Serra de Campos	Erva rupícola. Inflorescência com brácteas vermelhas. Frutos verdes com ápice roseo
Burmanniaceae	Burmannia	capitata	(Walter ex J.F.Gmel.) Mart.	Pastore, M.	644	2017	Brasil	Pará	São Félix do Xingu	Serra dos Carajás	Erva delicada. Flores creme-esverdeadas.
Burmanniaceae	Burmannia	flava	Mart.	Zappi, D.C.	3903	2018	Brasil	Pará	São Félix do Xingu	Serra de Campos, 20 km N de Tancredo Neves	Erva 15 cm compr., flores amarelas
Burmanniaceae	Burmannia	flava	Mart.	Viana, P.L.	6145	0	Brasil	Pará	São Félix do Xingu	Serra de Campos	
Burmanniaceae	Burmannia	flava	Mart.	Pastore, M.	615	2017	Brasil	Pará	São Félix do Xingu	Serra dos Carajás	Erva aquática. Flores amarelas.
Burmanniaceae	Burmannia	flava	Martius	Nogueira, M.G.C.	710	2017	Brasil	Pará	São Félix do Xingu	Serra de Campos	Erva sobre canga em solo úmido, flores amarelas.
Cabombaceae	Cabomba	furcata	Schult. & Schult.f.	Zappi, D.C.	3963	2018	Brasil	Pará	São Félix do Xingu	Serra de Campos, 20 km N de Tancredo Neves	Erva submersa, flores emersas rosa com centro amarelo
Commelinaceae	Commelina	erecta	L.	Zappi, D.C.	4058	2018	Brasil	Pará	São Félix do Xingu	Serra de Campos, 20 km N de Tancredo Neves	Erva 30 cm alt., em campo perturbado, flores azuis com estames amarelos
Commelinaceae	Dichorisandra	hexandra	(Aubl.) C.B. Clarke	Zappi, D.C.	3858	2018	Brasil	Pará	São Félix do Xingu	Serra de Campos, 20 km N de Tancredo Neves	Trepadeira sobre Fabaceae, pétalas azuis com a base alva, frutos atropurpureos
Commelinaceae	Dichorisandra	hexandra	(Aubl.) C.B. Clarke	Nogueira, M.G.C.	699	2017	Brasil	Pará	São Félix do Xingu	Serra de Campos	Trepadeira enrolando sobre árvore, folhas discolores, face superior verde mais escura, brilhantes, cálice verde, pétalas roxas com base branca.
Connaraceae	Rourea	ligulata	Baker	Andrino, C.O.	666	2019	Brasil	Pará	São Félix do Xingu	Serra de Campos	Arbusto escandente. Inflorescência com botões verdes. Frutos vermelhos, semente enegrecida
Convolvulaceae	Ipomoea	decora	Meisn.	Zappi, D.C.	4057	2018	Brasil	Pará	São Félix do Xingu	Serra de Campos, 20 km N de Tancredo Neves	Trepadeira, flores alvas com lobos magenta, pêndulas
Convolvulaceae	Distimake	macrocalyx	(Ruiz & Pav.) A.R. Simões & Staples	Pastore, M.	660	2017	Brasil	Pará	São Félix do Xingu	Serra dos Carajás	Trepadeira herbácea, ramos volúveis. Cálice verde esbranquiçado; corola branca; anteras espiralas amarelas, estigma branco.
Convolvulaceae	Ipomoea	marabaensis	D.F.Austin & Secco	Zappi, D.C.	3873	2018	Brasil	Pará	São Félix do Xingu	Serra de Campos, 20 km N de Tancredo Neves	Trepadeira com flores rosadas
Convolvulaceae	Ipomoea	marabaensis	D.F.Austin & Secco	Viana, P.L.	6108	0	Brasil	Pará	São Félix do Xingu	Serra de Campos	

Convolvulaceae	Ipomoea	marabaensis	D.F.Austin & Secco	Viana, P.L.	6110	0	Brasil	Pará	São Félix do Xingu	Serra de Campos	
Convolvulaceae	Ipomoea	marabaensis	D.F.Austin & Secco	Falcão, B.F.	618	2016	Brasil	Pará	São Félix do Xingu	Serra da Seringa	Erva escandente. Flores róseas, vistosas e estames insertos. Frutos secos abertos bege-claro. Pto. E3SER01JUN. DNA ITV2382
Convolvulaceae	Ipomoea	marabaensis	D.F.Austin & Secco	Nogueira, M.G.C.	701	2017	Brasil	Pará	São Félix do Xingu	Serra de Campos	Arbusto com ramos trepadores, folhas discolores, face superior verde mais escuro, pedúnculo e cálice creme vináceo, tubo roxo até o meio passando a lilás, fauce e tubo roxos, anteras brancas.
Convolvulaceae	Ipomoea	rubens	Choisy	Pastore, M.	672	2017	Brasil	Pará	São Félix do Xingu	próximo ao Rio Xin	Trepadeira herbácea sobre gramíneas, ramos volúveis. Cálice verde com indumento branco; corola rosa. ITV 1959.
Cyperaceae	Cyperus	aggregatus	(Willd.) Endl.	Zappi, D.C.	3865	2018	Brasil	Pará	São Félix do Xingu	Serra de Campos, 20 km N de Tancredo Neves	Erva, inflorescências castanho-avermelhadas
Cyperaceae	Rhynchospora	barbata	(Vahl) Kunth	Zappi, D.C.	3927	2018	Brasil	Pará	São Félix do Xingu	Serra de Campos, 20 km N de Tancredo Neves	Erva em touceiras, solo alagado, 30 cm alt., inflorescências castanhas
Cyperaceae	Rhynchospora	barbata	(Vahl) Kunth	Pastore, M.	652	2017	Brasil	Pará	São Félix do Xingu	Serra dos Carajás	Erva ereta, cespitosa. Espiguetas creme a castanhas, brácteas verdes.
Cyperaceae	Rhynchospora	barbata	(Vahl) Kunth	Andrino, C.O.	657	2019	Brasil	Pará	São Félix do Xingu	Serra de Campos	Erva, inflorescencia castanho claras.
Cyperaceae	Bulbostylis	conifera	(Kunth) C.B. Clarke	Zappi, D.C.	3895	2018	Brasil	Pará	São Félix do Xingu	Serra de Campos, 20 km N de Tancredo Neves	Touceiras com inflorescências ferrugíneas
Cyperaceae	Bulbostylis	conifera	(Kunth) C.B. Clarke	Viana, P.L.	6147	0	Brasil	Pará	São Félix do Xingu	Serra de Campos	
Cyperaceae	Bulbostylis	conifera	(Kunth) C.B. Clarke	Falcão, B.F.	616	2016	Brasil	Pará	São Félix do Xingu	Serra da Seringa	Erva rupícola. Inflorescência ferrugínea. Pto. E1SER01JUN. DNA ITV2384
Cyperaceae	Bulbostylis	conifera	(Kunth) C.B. Clarke	Falcão, B.F.	617	2016	Brasil	Pará	São Félix do Xingu	Serra da Seringa	Rupícola. Inflorescência ferrugínea. Pto. E2SER01JUN.
Cyperaceae	Bulbostylis	conifera	(Kunth) C.B. Clarke	Pastore, M.	650	2017	Brasil	Pará	São Félix do Xingu	Serra dos Carajás	Erva ereta com espiguetas castanhas a creme. ITV 1963.
Cyperaceae	Bulbostylis	conifera	(Kunth) C.B. Clarke	Nogueira, M.G.C.	716	2017	Brasil	Pará	São Félix do Xingu	Serra de Campos	Erva com espiguetas castanhas.
Cyperaceae	Bulbostylis	conifera	(Kunth) C.B. Clarke	Andrino, C.O.	624	2019	Brasil	Pará	São Félix do Xingu	Serra de Campos	Erva, espigueta castanha, filetes alvos.
Cyperaceae	Scleria	cyperina	Willd. ex Kunth	Zappi, D.C.	3925	2018	Brasil	Pará	São Félix do Xingu	Serra de Campos, 20 km N de Tancredo Neves	Erva crescendo entre arbustos, até 1 m alt., inflorescências jovens creme-esverdadas, velhas castanhas
Cyperaceae	Scleria	cyperina	Willd. ex Kunth	Falcão, B.F.	650	2016	Brasil	Pará	São Félix do Xingu	Serra da Seringa	Erva. Inflorescência esvedeada. Pto. E1SER11JUN. DNA ITV2394

Cyperaceae	Scleria	cyperina	Willd. ex Kunth	Pastore, M.	637	2017	Brasil	Pará	São Félix do Xingu	Serra dos Carajás	Erva até 1 m de altura. Inflorescência verde.
Cyperaceae	Rhynchospora	filiformis	Vahl	Zappi, D.C.	3930	2018	Brasil	Pará	São Félix do Xingu	Serra de Campos, 20 km N de Tancredo Neves	Erva crescendo em touceiras densas, inflorescências creme-esverdeadas
Cyperaceae	Rhynchospora	filiformis	Vahl	Viana, P.L.	6159	0	Brasil	Pará	São Félix do Xingu	Serra de Campos	
Cyperaceae	Rhynchospora	filiformis	Vahl	Pastore, M.	629	2017	Brasil	Pará	São Félix do Xingu	Serra dos Carajás	Erva associada à lateral da rocha, com espiguetas castanhas.
Cyperaceae	Rhynchospora	filiformis	Vahl	Pastore, M.	651	2017	Brasil	Pará	São Félix do Xingu	Serra dos Carajás	Erva cespitosa, ereta. Espiguetas castanhas.
Cyperaceae	Eleocharis	flavescens	(Poir.) Urb.	Pastore, M.	627	2017	Brasil	Pará	São Félix do Xingu	Serra dos Carajás	Erva aquática com espiguetas esverdeadas. ITV 1967.
Cyperaceae	Rhynchospora	holoschoenoides	(Rich.) Herter	Pastore, M.	608	2017	Brasil	Pará	São Félix do Xingu	Serra dos Carajás	Erva em campo brejoso. Capítulos castanhos; anteras creme
Cyperaceae	Diplasia	karatifolia	Rich. in Pers.	Zappi, D.C.	4032	2018	Brasil	Pará	São Félix do Xingu	Serra de Campos, 20 km N de Tancredo Neves	Erva aquática 1,5 m alt., frutos acastanhados
Cyperaceae	Cyperus	laxus	Lam.	Zappi, D.C.	3957	2018	Brasil	Pará	São Félix do Xingu	Serra de Campos, 20 km N de Tancredo Neves	Erva 50 cm alt., brácteas e inflorescências verde-amareladas
Cyperaceae	Scleria	microcarpa	Nees ex Kunth	Zappi, D.C.	3962	2018	Brasil	Pará	São Félix do Xingu	Serra de Campos, 20 km N de Tancredo Neves	Erva 1,5 m alt., inflorescências verde-claras
Cyperaceae	Scleria	microcarpa	Nees ex Kunth	Andrino, C.O.	650	2019	Brasil	Pará	São Félix do Xingu	Serra de Campos	
Cyperaceae	Eleocharis	pedrovianae	C.S. Nunes, R. Trevis. & A. Gil	Zappi, D.C.	4027	2018	Brasil	Pará	São Félix do Xingu	Serra de Campos, 20 km N de Tancredo Neves	Erva submersa, 10 cm alt.
Cyperaceae	Eleocharis	pedrovianae	C.S. Nunes, R. Trevis. & A. Gil	Viana, P.L.	6174	0	Brasil	Pará	São Félix do Xingu	Serra de Campos	
Cyperaceae	Eleocharis	pedrovianae	C.S. Nunes, R. Trevis. & A. Gil	Falcão, B.F.	628	2016	Brasil	Pará	São Félix do Xingu	Serra da Seringa	Erva aquática. Altura 15 cm. Inflorescência amarronzada.. Pto. E3SER02JUN.
Cyperaceae	Eleocharis	pedrovianae	C.S. Nunes, R. Trevis. & A. Gil	Falcão, B.F.	643	2016	Brasil	Pará	São Félix do Xingu	Serra da Seringa	Erva terrestre. Inflorescência amarronzada. Pto. E1SER08JUN.
Cyperaceae	Eleocharis	pedrovianae	C.S. Nunes, R. Trevis. & A. Gil	Pastore, M.	623	2017	Brasil	Pará	São Félix do Xingu	Serra dos Carajás	Erva sobre canga úmida.
Cyperaceae	Eleocharis	pedrovianae	C.S. Nunes, R. Trevis. & A. Gil	Pastore, M.	631	2017	Brasil	Pará	São Félix do Xingu	Serra dos Carajás	Erva aquática, totalmente submersa, associada a Eleocharis.
Cyperaceae	Eleocharis	plicarhachis	(Griseb.) Svenson	Andrino, C.O.	678	2019	Brasil	Pará	São Félix do Xingu	Serra de Campos	Erva na lagoa. Inflorescência verde
Cyperaceae	Rhynchospora	seccoi	C.S.Nunes, P.J.S. Silva Filho & A.Gil	Zappi, D.C.	3905	2018	Brasil	Pará	São Félix do Xingu	Serra de Campos, 20 km N	Erva 15 cm alt., flores esverdeadas

										de Tancredo Neves	
Cyperaceae	Rhynchospora	seccoi	C.S.Nunes, P.J.S. Silva Filho & A.Gil	Pastore, M.	621	2017	Brasil	Pará	São Félix do Xingu	Serra dos Carajás	Erva sobre canga, associada com briófitas.
Cyperaceae	Rhynchospora	seccoi	C.S.Nunes, P.J.S. Silva Filho & A.Gil	Pastore, M.	630	2017	Brasil	Pará	São Félix do Xingu	Serra dos Carajás	Erva associada à lateral da rocha, com espiguetas castanhas.
Cyperaceae	Rhynchospora	seccoi	C.S.Nunes, P.J.S. Silva Filho & A.Gil	Pastore, M.	643	2017	Brasil	Pará	São Félix do Xingu	Serra dos Carajás	Erva sobre briófitas. Brácteas castanhas. ITV 1964.
Cyperaceae	Cyperus	sesquiflorus	(Torr.) Mattf. & Kük.	Zappi, D.C.	4031	2018	Brasil	Pará	São Félix do Xingu	Serra de Campos, 20 km N de Tancredo Neves	Erva em solo encharcado, 10 cm alt., inflorescências creme-esverdeadas
Cyperaceae	Cyperus	sphacelatus	Rottb.	Zappi, D.C.	4042	2018	Brasil	Pará	São Félix do Xingu	Serra de Campos, 20 km N de Tancredo Neves	Erva 30 cm alt., inflorescências verde-claras
Dennstaedtiaceae	Pteridium	arachnoideum	(Kauf.) Maxon	Zappi, D.C.	4002	2018	Brasil	Pará	São Félix do Xingu	Serra de Campos, 20 km N de Tancredo Neves	Samambaia muito comum ocupando o limite entre a floresta e a canga
Dioscoreaceae	Dioscorea	piperifolia	Humb. & Bonpl. ex Willd.	Zappi, D.C.	3884	2018	Brasil	Pará	São Félix do Xingu	Serra de Campos, 20 km N de Tancredo Neves	Trepadeira, inflorescências esverdeadas, folhas dimórficas
Dioscoreaceae	Dioscorea	piperifolia	Humb. & Bonpl. ex Willd.	Zappi, D.C.	4018	2018	Brasil	Pará	São Félix do Xingu	Serra de Campos, 20 km N de Tancredo Neves	Erva trepadeira, flores creme-amareladas
Dioscoreaceae	Dioscorea	trilinguis	Griseb.	Zappi, D.C.	3934	2018	Brasil	Pará	São Félix do Xingu	Serra de Campos, 20 km N de Tancredo Neves	Trepadeira com frutos velhos castanhos
Eriocaulaceae	Eriocaulon	carajense	Moldenke	Zappi, D.C.	3936	2018	Brasil	Pará	São Félix do Xingu	Serra de Campos, 20 km N de Tancredo Neves	Erva aquática, capítulos alvos
Eriocaulaceae	Eriocaulon	carajense	Moldenke	Viana, P.L.	6164	0	Brasil	Pará	São Félix do Xingu	Serra de Campos	
Eriocaulaceae	Eriocaulon	carajense	Moldenke	Viana, P.L.	6171	0	Brasil	Pará	São Félix do Xingu	Serra de Campos	
Eriocaulaceae	Eriocaulon	carajense	Moldenke	Falcão, B.F.	621	2016	Brasil	Pará	São Félix do Xingu	Serra da Seringa	Erva terrestre. Inflorescência branca. Pto. E2SER02JUN.

Eriocaulaceae	Eriocaulon	carajense	Moldenke	Pastore, M.	620	2017	Brasil	Pará	São Félix do Xingu	Serra dos Carajás, Serra de Campos	Erva rara, em canga úmida. Brácteas creme; anteras negras. ITV 1969.
Eriocaulaceae	Eriocaulon	carajense	Moldenke	Pastore, M.	648	2017	Brasil	Pará	São Félix do Xingu	Serra dos Carajás, Serra de Campos	Erva robusta, com muitos escapos, as vezes com base do escapo rosa, formando grande população. Capítulos brancos; anteras negras. (Foto de Ray Harley). ITV 1950.
Eriocaulaceae	Eriocaulon	cinereum	R.Br.	Zappi, D.C.	4049	2018	Brasil	Pará	São Félix do Xingu	Serra de Campos, 20 km N de Tancredo Neves	Erva aquática, capítulos creme
Eriocaulaceae	Eriocaulon	cinereum	R.Br.	Pastore, M.	622	2017	Brasil	Pará	São Félix do Xingu	Serra dos Carajás	Erva ereta, capítulos brancos.
Eriocaulaceae	Syngonanthus	discretifolius	(Moldenke) M.T.C. Watanabe	Viana, P.L.	6119	2016	Brasil	Pará	São Félix do Xingu	Serra de Campos	Erva anual em área úmida. Capítulos brancos.
Eriocaulaceae	Syngonanthus	discretifolius	(Moldenke) M.T.C. Watanabe	Viana, P.L.	6140	2016	Brasil	Pará	São Félix do Xingu	Serra de Campos	Erva de capítulos brancos
Eriocaulaceae	Syngonanthus	discretifolius	(Moldenke) M.T.C. Watanabe	Viana, P.L.	6168	2016	Brasil	Pará	São Félix do Xingu	Serra de Campos	Erva anual com capítulos brancos
Eriocaulaceae	Syngonanthus	discretifolius	(Moldenke) M.T.C.Watanabe	Falcão, B.F.	622	2016	Brasil	Pará	São Félix do Xingu	Serra da Seringa	Terrícola. Inflorescência branca. Pto. E2SER02JUN.
Eriocaulaceae	Syngonanthus	discretifolius	(Moldenke) M.T.C.Watanabe	Pastore, M.	642	2017	Brasil	Pará	São Félix do Xingu	Serra dos Carajás	Erva, indivíduos jovens. Capítulos brancos. ITV 1960.
Eriocaulaceae	Paepalanthus	fasciculoides	Hensold	Zappi, D.C.	3878	2018	Brasil	Pará	São Félix do Xingu	Serra de Campos, 20 km N de Tancredo Neves	Erva com brácteas enegrecidas, capítulos alvos
Eriocaulaceae	Paepalanthus	fasciculoides	Hensold	Viana, P.L.	6104	0	Brasil	Pará	São Félix do Xingu	Serra de Campos	
Eriocaulaceae	Paepalanthus	fasciculoides	Hensold	Viana, P.L.	6141	0	Brasil	Pará	São Félix do Xingu	Serra de Campos	
Eriocaulaceae	Paepalanthus	fasciculoides	Hensold	Viana, P.L.	6173	0	Brasil	Pará	São Félix do Xingu	Serra de Campos	
Eriocaulaceae	Paepalanthus	fasciculoides	Hensold	Pastore, M.	663	2017	Brasil	Pará	São Félix do Xingu	Serra dos Carajás	Erva com capítulos acinzentados. ITV 1958.
Eriocaulaceae	Paepalanthus	fasciculoides	Hensold	Nogueira, M.G.C.	698	2017	Brasil	Pará	São Félix do Xingu	Serra de Campos	Erva sobre canga, capítulos castanhos, antera creme.
Eriocaulaceae	Syngonanthus	heteropeplus	Ruhland	Viana, P.L.	6170	0	Brasil	Pará	São Félix do Xingu	Serra de Campos	
Eriocaulaceae	Syngonanthus	heteropeplus	(Körn.) Ruhland	Pastore, M.	659	2017	Brasil	Pará	São Félix do Xingu	Serra dos Carajás	Erva com folhas as vezes cobertas pela água. Capítulos com brácteas e flores creme, escapos verdes.
Erythroxylacaeae	Erythroxylum	nelson-rosae	Plowman	Zappi, D.C.	3940	2018	Brasil	Pará	São Félix do Xingu	Serra de Campos, 20 km N de Tancredo Neves	Arbusto 2,5 m alt., estéril
Erythroxylaceae	Erythroxylum	nelson-rosae	Plowman	Andrino, C.O.	672	2019	Brasil	Pará	São Félix do Xingu	Serra de Campos	Arbusto, 2 m alt. estéril.

Erythroxylaceae	Erythroxylum	rufum	Cav.				Zappi, D.C.	3889	2018	Brasil	Pará	São Félix do Xingu	Serra de Campos, 20 km N de Tancredo Neves	Arbusto 1,6 m alt., estéril
Erythroxylaceae	Erythroxylum	rufum	Cav.				Zappi, D.C.	4054	2018	Brasil	Pará	São Félix do Xingu	Serra de Campos, 20 km N de Tancredo Neves	Árvore 6 m alt., na borda do capão, estéril
Erythroxylaceae	Erythroxylum	rufum	Cav.				Andrino, C.O.	637	2019	Brasil	Pará	São Félix do Xingu	Serra de Campos	Árvore de 3,5. Folhas discolores. Frutos verdes. ITV21710
Euphorbiaceae	Mabea	angustifolia	Spruce ex Benth.				Zappi, D.C.	3987	2018	Brasil	Pará	São Félix do Xingu	Serra de Campos, 20 km N de Tancredo Neves	Arbusto 4 m alt., inflorescências pendentes verde-avermelhadas
Euphorbiaceae	Maprounea	brasiliensis	A.St.-Hil.				Zappi, D.C.	3991	2018	Brasil	Pará	São Félix do Xingu	Serra de Campos, 20 km N de Tancredo Neves	Arbusto 3 m alt., estéril
Euphorbiaceae	Aparisthmium	cordatum	(A. Juss.) Baill.				Zappi, D.C.	3997	2018	Brasil	Pará	São Félix do Xingu	Serra de Campos, 20 km N de Tancredo Neves	Árvore 6 m alt., inflorescências esverdeadas
Euphorbiaceae	Alchornea	discolor	Poeppig				Zappi, D.C.	3886	2018	Brasil	Pará	São Félix do Xingu	Serra de Campos, 20 km N de Tancredo Neves	Arbusto a arvoreta 2 m alt., estéril
Euphorbiaceae	Alchornea	discolor	Poeppig				Andrino, C.O.	631	2019	Brasil	Pará	São Félix do Xingu	Serra de Campos	Arvoreta 3 m. Folhas discolores. Frutos verdes a vináceos.
Euphorbiaceae	Astraea	lobata	(L.) Klotzsch				Zappi, D.C.	3955	2018	Brasil	Pará	São Félix do Xingu	Serra de Campos, 20 km N de Tancredo Neves	Arbusto delicado, inflorescências avermelhadas, flores creme
Euphorbiaceae	Astraea	lobata	(L.) Klotzsch				Pastore, M.	611	2017	Brasil	Pará	São Félix do Xingu	Serra dos Carajás	Subarbusto ca. 80 cm alt., ramos avermelhados. Cálice verde a vináceo; corola creme. Flores femininas com ovário verde, estiletes vermelhos.
Euphorbiaceae	Manihot	quinquepartita	Huber ex D.J.Rogers				Zappi, D.C.	3954	2018	Brasil	Pará	São Félix do Xingu	Serra de Campos, 20 km N de Tancredo Neves	Arbusto latescente, esgalhado 4 m alt.., botões azulados, flores amarelas campanuladas
Euphorbiaceae	Manihot	tristis	Müll.Arg.	subsp	surumuensis	(Ule) D.J.Rogers & Appan	Pastore, M.	666	2017	Brasil	Pará	São Félix do Xingu	Serra dos Carajás	Arbusto ca. 1,5 m alt. Cálice verde com estria vinácea. Frutos verdes com estrias vináceas.

Fabaceae	Dioclea	apurensis	Kunth	Zappi, D.C.	3919	2018	Brasil	Pará	São Félix do Xingu	Serra de Campos, 20 km N de Tancredo Neves	Trepadeira robusta, sobre arbustos, flores violáceas com interior mais claro
Fabaceae	Dioclea	apurensis	Kunth	Andrino, C.O.	662	2019	Brasil	Pará	São Félix do Xingu	Serra de Campos	Trepadeira, frutos castanhos.
Fabaceae	Inga	calantha	Ducke	Zappi, D.C.	3986	2018	Brasil	Pará	São Félix do Xingu	Serra de Campos, 20 km N de Tancredo Neves	Árvore 3 m alt., esteril
Fabaceae	Inga	calantha	Ducke	Andrino, C.O.	600	2019	Brasil	Pará	São Félix do Xingu	Serra de Campos	Arvoreta até 5 m., folhas discolores, fruto castanho esverdeado.
Fabaceae	Stylosanthes	capitata	Vogel	Zappi, D.C.	3977	2018	Brasil	Pará	São Félix do Xingu	Serra de Campos, 20 km N de Tancredo Neves	Subarbusto esgalhado 60 cm alt., flores amarelas
Fabaceae	Centrosema	carajasense	Cavalcante,	Zappi, D.C.	4007	2018	Brasil	Pará	São Félix do Xingu	Serra de Campos, 20 km N de Tancredo Neves	Trepadeira delicada, estéril
Fabaceae	Centrosema	carajasense	Cavalc.	Falcão, B.F.	636	2016	Brasil	Pará	São Félix do Xingu	Serra da Seringa	Erva escandente. Fruto verde. Pto. E1SER11JUN. DNA ITV2396
Fabaceae	Mimosa	dasilvae	A.S.L. Silva & Secco	Zappi, D.C.	3897	2018	Brasil	Pará	São Félix do Xingu	Serra de Campos, 20 km N de Tancredo Neves	Subarbusto com ramos rasteiros, flores rosadas, frutos verde-dourados
Fabaceae	Mimosa	dasilvae	A.S.L. Silva & Secco	Viana, P.L.	6106	0	Brasil	Pará	São Félix do Xingu	Serra de Campos	
Fabaceae	Mimosa	dasilvae	A.S.L. Silva & Secco	Viana, P.L.	6157	0	Brasil	Pará	São Félix do Xingu	Serra de Campos	
Fabaceae	Mimosa	dasilvae	A.S.L. Silva & Secco	Silva, J.B.F. da	501	1995	Brasil	Pará	São Félix do Xingu	São Felix do Xingu	Planta rasteira tipo liana com mais de 10m de comprimento (não sobe), crescendo em campo rupestre aberto, entre rocha tipo canga. Inflorescência terminal com flores cor de rosa, botões esverdeados, mu
Fabaceae	Mimosa	dasilvae	A.S.Silva & R.Secco	Falcão, B.F.	646	2016	Brasil	Pará	São Félix do Xingu	Serra da Seringa	Erva terrestre. Frutos marrons.. Pto. E1SER10JUN.
Fabaceae	Mimosa	dasilvae	A.S.L. Silva & Secco	Nogueira, M.G.C.	719	2017	Brasil	Pará	São Félix do Xingu	Serra de Campos	Arbusto com cerca de 60 cm de alt., frutos verdes.
Fabaceae	Mimosa	dasilvae	A.S.L. Silva & Secco	Andrino, C.O.	622	2019	Brasil	Pará	São Félix do Xingu	Serra de Campos	Arbusto, estames róseos.
Fabaceae	Chamaecrista	desvauxii	(Collad.) Killip	Zappi, D.C.	3946	2018	Brasil	Pará	São Félix do Xingu	Serra de Campos, 20 km N de Tancredo Neves	Subarbusto 30 cm alt., flores amarelas
Fabaceae	Camptosema	ellipticum	(Desv.) Burkart	Zappi, D.C.	3907	2018	Brasil	Pará	São Félix do Xingu	Serra de Campos,	Arbusto 2 m alt., flores vermelhas

													20 km N de Tancredo Neves	
Fabaceae	Camptosema	ellipticum	(Desv.) Burkart				Nogueira, M.G.C.	722	2017	Brasil	Pará	São Félix do Xingu	Serra de Campos	Trepadeira, cálice e corola com tom avermelhado.
Fabaceae	Clitoria	falcata	Lam.	var.	falcata		Zappi, D.C.	3917	2018	Brasil	Pará	São Félix do Xingu	Serra de Campos, 20 km N de Tancredo Neves	Trepadeira rasteira, flores alvas, estandarte com estrias arroxeadas, fruto verde
Fabaceae	Clitoria	falcata	Lam.	var.	falcata		Pastore, M.	657	2017	Brasil	Pará	São Félix do Xingu	Serra dos Carajás	Trepadeira volúvel. Cálice verde; corola branca com linhas roxas no estandarte.
Fabaceae	Mimosa	guilandinae		var.	spruceana	(Benth.) Barneby	Andrino, C.O.	668	2019	Brasil	Pará	São Félix do Xingu	Serra de Campos	Arbusto ca. 1 m alt. Folhas discolores. Frutos castanhos.
Fabaceae	Inga	heterophylla	Willd				Zappi, D.C.	4036	2018	Brasil	Pará	São Félix do Xingu	Serra de Campos, 20 km N de Tancredo Neves	Árvore 6 m alt. na beira do capão, botões verdes
Fabaceae	Inga	leiocalycina	Benth.				Pastore, M.	598	2017	Brasil	Pará	São Félix do Xingu	a caminho da Serra	Árvore ca. 5 m alt. Folhas levemente discolores. Cálice e corola verdes. Filetes brancos e anteras amarelas.
Fabaceae	Crotalaria	maypurensis	Kunth				Zappi, D.C.	3881	2018	Brasil	Pará	São Félix do Xingu	Serra de Campos, 20 km N de Tancredo Neves	Arbusto 50 cm alt., flores amarelas, frutos verdes
Fabaceae	Crotalaria	maypurensis	Kunth				Pastore, M.	625	2017	Brasil	Pará	São Félix do Xingu	Serra dos Carajás	Erva até 1 m alt. Folhas discolores. Flores amarelas. Frutos verdes.
Fabaceae	Crotalaria	maypurensis	Kunth				Nogueira, M.G.C.	706	2017	Brasil	Pará	São Félix do Xingu	Serra de Campos	Erva até 60 cm de altura, folhas discolores com a face superior verde mais escura, cálice amarelo com tons vináceos, pétalas amarelas, estandarte com face inferior em tom castanho. Fruto verde.
Fabaceae	Periandra	mediterranea	(Vell.) Taub.				Zappi, D.C.	3902	2018	Brasil	Pará	São Félix do Xingu	Serra de Campos, 20 km N de Tancredo Neves	Arbusto 1 m alt., flores azuladas
Fabaceae	Periandra	mediterranea	(Vell.) Taub.				Nogueira, M.G.C.	717	2017	Brasil	Pará	São Félix do Xingu	Serra de Campos	Trepadeira de flores azuis.
Fabaceae	Abrus	melanospermus	Hassk.	subsp.	tenuiflorus	(Spruce ex Benth.) D. Harder	Zappi, D.C.	3912	2018	Brasil	Pará	São Félix do Xingu	Serra de Campos, 20 km N de Tancredo Neves	Trepadeira delicada, flores lilases, estandarte com estrias creme
Fabaceae	Abrus	melanospermus	Hassk.	subsp	tenuiflorus	(Spruce ex Benth.) D. Harder	Pastore, M.	616	2017	Brasil	Pará	São Félix do Xingu	Serra dos Carajás	Trepadeira herbácea sobre gramínea. Corola lilás com o centro amarelado.
Fabaceae	Senegalia	multipinnata	(Ducke) Seigler & Ebinger				Andrino, C.O.	603	2019	Brasil	Pará	São Félix do Xingu	Serra de Campos	Arvoreta até 6 metros, estames brancos.

Fabaceae	Parkia	platycephala	Benth.				Zappi, D.C.	4013	2018	Brasil	Pará	São Félix do Xingu	Serra de Campos, 20 km N de Tancredo Neves	Arbusto 4 m alt., estéril
Fabaceae	Bauhinia	pulchella	Benth.				Zappi, D.C.	3869	2018	Brasil	Pará	São Félix do Xingu	Serra de Campos, 20 km N de Tancredo Neves	Arbusto 1,5 m alt., botões ferrugíneos, estames alvos
Fabaceae	Bauhinia	pulchella	Benth.				Viana, P.L.	6121	0	Brasil	Pará	São Félix do Xingu	Serra de Campos	
Fabaceae	Bauhinia	pulchella	Benth.				Nogueira, M.G.C.	728	2017	Brasil	Pará	São Félix do Xingu	Serra de Campos	Arbusto com cerca de 2,5 m de altura, cálice castanho, pétalas e estames brancos.
Fabaceae	Aeschynomene	sensitiva		var.	hispidula		Zappi, D.C.	4024	2018	Brasil	Pará	São Félix do Xingu	Serra de Campos, 20 km N de Tancredo Neves	Subarbusto com ramos basais inflados e verrucosos, aquática, 40 cm compr., flores amarelas com estrias mais escuras
Fabaceae	Aeschynomene	sensitiva		var.	hispidula		Andrino, C.O.	675	2019	Brasil	Pará	São Félix do Xingu	Serra de Campos	Arbusto 1 m alt. Flores amarelos, estandarte com nervuras castanhas
Fabaceae	Mimosa	skinneri	Benth.				Zappi, D.C.	3860	2018	Brasil	Pará	São Félix do Xingu	Serra de Campos, 20 km N de Tancredo Neves	Erva espinhosa, ramos avermelhados, flores alvas
Fabaceae	Mimosa	skinneri	Benth.	var.	arajarum	Barneby	Falcão, B.F.	619	2016	Brasil	Pará	São Félix do Xingu	Serra da Seringa	Erva terrestre. Frutos verdes, erva prostada ao solo.. Pto. E1SER01JUN.
Fabaceae	Mimosa	skinneri	Benth	var.	carajarum	Barneby	Falcão, B.F.	623	2016	Brasil	Pará	São Félix do Xingu	Serra da Seringa	Erva terrestre. Frutos verdes, erva prostada ao solo.. Pto. E3SER02JUN.
Fabaceae	Mimosa	skinneri	Benth.	var.	carajarum	Barneby	Nogueira, M.G.C.	738	2017	Brasil	Pará	São Félix do Xingu	Serra de Campos	Erva terrestre.
Fabaceae	Mimosa	somnians	Humb. & Bonpl. ex Willd.	var.	viscida	(Willd.) Barneby	Zappi, D.C.	3876	2018	Brasil	Pará	São Félix do Xingu	Serra de Campos, 20 km N de Tancredo Neves	Subarbusto rasteiro, folíolos sensitivos, flores rosadas com anteras amarelas
Fabaceae	Mimosa	somnians	Humb. & Bonpl. ex Willd.				Zappi, D.C.	3958	2018	Brasil	Pará	São Félix do Xingu	Serra de Campos, 20 km N de Tancredo Neves	Arbusto espinescente 2 m alt., ramos avermelhados, inflorescências passadas rosadas
Fabaceae	Mimosa	somnians	Humb. & Bonpl. ex Willd.	var.	viscida	(Willd.) Barneby	Falcão, B.F.	647	2016	Brasil	Pará	São Félix do Xingu	Serra da Seringa	Subarbusto. Flor rosa.. Pto. E1SER11JUN.
Fabaceae	Mimosa	somnians	Humb. & Bonpl. ex Willd.	var.	viscida	(Willd.) Barneby	Nogueira, M.G.C.	746	2017	Brasil	Pará	São Félix do Xingu	Serra de Campos	Erva até 1 m de altura, caule com indumento castanho, lâminas verdes, discolores, estilete róseo.
Fabaceae	Tachigali	vulgaris	L.F.G.Silva & H.C.Lima				Andrino, C.O.	655	2019	Brasil	Pará	São Félix do Xingu	Serra de Campos	Árvore 10 m. Frutos castanhos.
Fabaceae	Mimosa	xanthocentra	Mart.	var.	mansii	(Mart.) Barneby	Viana, P.L.	6158	0	Brasil	Pará	São Félix do Xingu	Serra de Campos	
Gentianaceae	Schultesia	benthamiana	Klotzsch ex Griseb.				Zappi, D.C.	3928	2018	Brasil	Pará	São Félix do Xingu	Serra de Campos,	Erva 15 cm alt., delicada, cálice verde, flores amarelo-alaranjadas

											20 km N de Tancredo Neves	
Heliconiaceae	Heliconia		psittacorum	L.f.	Pastore, M.	671	2017	Brasil	Pará	São Félix do Xingu	Serra dos Carajás	Erva ca. 1 m alt. Folhas discolores. Brácteas amarelas e pétalas amarelas com o ápice verde.
Hypericaceae	Vismia		gracilis	Hieron	Andrino, C.O.	654	2019	Brasil	Pará	São Félix do Xingu	Serra de Campos	Árvore 4 m. Folhas discolores, face abaxial argentea. Frutos verdes.
Iridaceae	Cipura		xanthomelas	Maxim. ex Klatt	Zappi, D.C.	3899	2018	Brasil	Pará	São Félix do Xingu	Serra de Campos, 20 km N de Tancredo Neves	Erva 50 cm compr., flores amarelas, frutos verdes
Lamiaceae	Hyptis		atrorubens	Poit.	Zappi, D.C.	3981	2018	Brasil	Pará	São Félix do Xingu	Serra de Campos, 20 km N de Tancredo Neves	Erva rasteira 20 cm alt., glomérulos verde-claros, corola creme
Lamiaceae	Amasonia		lasiocaulos	Mart. & Schau ex Schau.	Zappi, D.C.	3947	2018	Brasil	Pará	São Félix do Xingu	Serra de Campos, 20 km N de Tancredo Neves	Subarbusto 50 cm alt., inflorescências vermelhas, flores amarelo-claras
Lamiaceae	Vitex		panshiniana	Moldenke	Zappi, D.C.	4053	2018	Brasil	Pará	São Félix do Xingu	Serra de Campos, 20 km N de Tancredo Neves	Árvore 6 m alt., na borda do capão, estéril
Lamiaceae	Mesosphaerum		pectinatum	(L.) Kuntze	Nogueira, M.G.C.	697	2017	Brasil	Pará	São Félix do Xingu	Serra de Campos	Erva até 1,5 m, inflorescência com muitas flores.
Lamiaceae	Mesosphaerum		suaveolens	(L.) Kuntze	Zappi, D.C.	4048	2018	Brasil	Pará	São Félix do Xingu	Serra de Campos, 20 km N de Tancredo Neves	Erva 1 m alt., em campo perturbado, flores azuis, apenas foto
Lamiaceae	Mesosphaerum		suaveolens	(L.) Kuntze	Nogueira, M.G.C.	696	2017	Brasil	Pará	São Félix do Xingu	Serra de Campos	Erva até 1,5 m, inflorescência com poucas flores.
Lauraceae	Dicypellium	aff.	caryophyllaceum	(Mart.) Nees	Viana, P.L.	6100	0	Brasil	Pará	São Félix do Xingu	Serra de Campos	
Lauraceae	Cassytha		filiformis	L.	Zappi, D.C.	3874	2018	Brasil	Pará	São Félix do Xingu	Serra de Campos, 20 km N de Tancredo Neves	Parasita, flores alvas, frutos verde-amarelados
Lauraceae	Cassytha		filiformis	L.	Viana, P.L.	6102	2016	Brasil	Pará	São Félix do Xingu	Serra de Campos	Parasita, ramos esverdiados. Frutos imaturos verdes.
Lauraceae	Cassytha		filiformis	L.	Nogueira, M.G.C.	715	2017	Brasil	Pará	São Félix do Xingu	Serra de Campos, estrada no interior da Serra de Campos.	Parasita, ramos verdes a amarelados, corola amarela.

Lauraceae	Mezilaurus	itauba	(Meisn.) Taub. ex Mez	Zappi, D.C.	4001	2018	Brasil	Pará	São Félix do Xingu	Serra de Campos, 20 km N de Tancredo Neves	Arbusto crescendo à sombra, botões alvos
Lauraceae	Rhodostemonodaphne	praeclara	(Sandwith) Madriñán	Zappi, D.C.	3983	2018	Brasil	Pará	São Félix do Xingu	Serra de Campos, 20 km N de Tancredo Neves	Árvore 5 m alt., flores creme, anteras amarelas
Lauraceae	Dicypellium	sp nov		Andrino, C.O.	633	2019	Brasil	Pará	São Félix do Xingu	Serra de Campos	Arbusto até 2 m. Flores alvas.
Lentibulariaceae	Utricularia	neottioides	A.St-Hil & Girard	Pastore, M.	664	2017	Brasil	Pará	São Félix do Xingu	Serra dos Carajás, Serra de Campos	Erva ereta. Flores brancas a esverdeadas.
Lentibulariaceae	Utricularia	pusilla	Vahl	Zappi, D.C.	3904	2018	Brasil	Pará	São Félix do Xingu	Serra de Campos, 20 km N de Tancredo Neves	Erva 10 cm compr., flores amarelas
Lentibulariaceae	Utricularia	pusilla	Vahl	Zappi, D.C.	3943	2018	Brasil	Pará	São Félix do Xingu	Serra de Campos, 20 km N de Tancredo Neves	Erva aquática 5 cm alt., flores amarelas
Lentibulariaceae	Utricularia	pusilla	Vahl	Viana, P.L.	6138	2016	Brasil	Pará	São Félix do Xingu	Serra de Campos	Flores amarelas
Lentibulariaceae	Utricularia	pusilla	Vahl	Viana, P.L.	6163	2016	Brasil	Pará	São Félix do Xingu	Serra de Campos	Flores amarelas, em área brejosa
Lentibulariaceae	Utricularia	pusilla	Vahl	Pastore, M.	649	2017	Brasil	Pará	São Félix do Xingu	Serra dos Carajás, Serra de Campos	Erva com ramos delgados. Flores com corola toda amarela e flores com o centro da corola amarelo e lobos creme.
Lentibulariaceae	Utricularia	pusilla	Vahl	Nogueira, M.G.C.	705	2017	Brasil	Pará	São Félix do Xingu	Serra de Campos	Erva em local úmido, flores amarelas.
Lentibulariaceae	Utricularia	subulata	L.	Viana, P.L.	6139	2016	Brasil	Pará	São Félix do Xingu	Serra de Campos	Flores amarelas
Loranthaceae	Psittacanthus	eucalyptifolius	(Kunth) G. Don	Zappi, D.C.	4056	2018	Brasil	Pará	São Félix do Xingu	Serra de Campos, 20 km N de Tancredo Neves	Parasita com frutos vermelho-esverdeados
Loranthaceae	Passovia	pedunculata	(Jacq.) Kuijt	Zappi, D.C.	3909	2018	Brasil	Pará	São Félix do Xingu	Serra de Campos, 20 km N de Tancredo Neves	Parasita, flores alvas com anteras amarelas, frutos verdes
Loranthaceae	Passovia	pedunculata	(Jacq.) Kuijt	Nogueira, M.G.C.	747	2017	Brasil	Pará	São Félix do Xingu	Serra de Campos	Arbusto até 1,5 m de altura, lâminas verdes, concolores, frutos com base verde e o restante laranja-avermelhados.
Loranthaceae	Passovia	pedunculata	(Jacq.) Kuijt	Andrino, C.O.	648	2019	Brasil	Pará	São Félix do Xingu	Serra de Campos	Erva parasita, flores alvas.

Lythraceae	Cuphea	annulata	Koehne	Zappi, D.C.	3864	2018	Brasil	Pará	São Félix do Xingu	Serra de Campos, 20 km N de Tancredo Neves	Subarbusto lenhoso apenas na base, flores viscosas, pétalas rosa-magenta
Lythraceae	Cuphea	annulata	Koehne	Viana, P.L.	6105	2016	Brasil	Pará	São Félix do Xingu	Serra de Campos	Flores com cálice alaranjado. Corola inconspícua.
Lythraceae	Cuphea	annulata	Koehne	Pastore, M.	639	2017	Brasil	Pará	São Félix do Xingu	Serra dos Carajás, Serra de Campos	Subarbusto ca. 40 cm alt.. Tubo floral creme-avermelhado a laranjado.
Lythraceae	Cuphea	annulata	Koehne	Nogueira, M.G.C.	703	2017	Brasil	Pará	São Félix do Xingu	Serra de Campos	Arbusto muito ramificado, cerca de 60 cm de altura, folhas discolores, face superior verde mais escuro, cálice com tubo amarelo e avermelhado no ápice, pétalas brancas.
Lythraceae	Cuphea	carajasensis	Lourteig	Zappi, D.C.	3870	2018	Brasil	Pará	São Félix do Xingu	Serra de Campos, 20 km N de Tancredo Neves	Arbusto esgalhado, hipanto amarelo-alaranjado
Lythraceae	Cuphea	carajasensis	Lourteig	Viana, P.L.	6136	2016	Brasil	Pará	São Félix do Xingu	Serra de Campos	Subarbusto com flores roxas
Lythraceae	Cuphea	carajasensis	Lourteig	Viana, P.L.	6116	2016	Brasil	Pará	São Félix do Xingu	Serra de Campos	Arbusto até 50 cm. Flores com pétalas lilases.
Lythraceae	Cuphea	carajasensis	Lourteig	Pastore, M.	618	2017	Brasil	Pará	São Félix do Xingu	Serra dos Carajás	Erva até 50 cm alt. Cálice verde; corola lilás. ITV 1966.
Lythraceae	Cuphea	carajasensis	Lourteig	Nogueira, M.G.C.	709	2017	Brasil	Pará	São Félix do Xingu	Serra de Campos	Subarbusto até 30 cm, muito ramificado, pétalas magenta.
Lythraceae	Cuphea	carajasensis	Lourteig	Andrino, C.O.	616	2019	Brasil	Pará	São Félix do Xingu	Serra de Campos	Arbusto 40 cm. Corola rosa.
Malpighiaceae	Byrsonima	chrysophylla	Kunth	Zappi, D.C.	3929	2018	Brasil	Pará	São Félix do Xingu	Serra de Campos, 20 km N de Tancredo Neves	Arbusto muito comum na área, 0,5-2 m alt., frutos verdes passando a amarelos
Malpighiaceae	Byrsonima	chrysophylla	Kunth	Pastore, M.	662	2017	Brasil	Pará	São Félix do Xingu	Serra dos Carajás	Arbusto até 2 m alt. Folhas discolores. Cálice verde a castanho; corola amarela; anteras castanhas.
Malpighiaceae	Banisteriopsis	malifolia	(Nees & Mart.) B.Gates	Nogueira, M.G.C.	743	2017	Brasil	Pará	São Félix do Xingu	Serra de Campos	Arbusto até 1,5 m, folhas discolores com indumento esbraquiçado na face inferior, cálice verde e corola branca. Botões verdes, estilete verde e estigma amarelo esverdeado.
Malpighiaceae	Heteropterys	nervosa	A.Juss.	Andrino, C.O.	645	2019	Brasil	Pará	São Félix do Xingu	Serra de Campos	Trepadeira, frutos verde claros.
Malpighiaceae	Banisteriopsis	stellaris	(Griseb.) B.Gates	Zappi, D.C.	3863	2018	Brasil	Pará	São Félix do Xingu	Serra de Campos, 20 km N de Tancredo Neves	Trepadeira, botões amarelados, pétalas creme
Malpighiaceae	Banisteriopsis	stellaris	(Griseb.) B.Gates	Viana, P.L.	6107	0	Brasil	Pará	São Félix do Xingu	Serra de Campos	
Malpighiaceae	Banisteriopsis	stellaris	(Griseb.) B.Gates	Pastore, M.	606	2017	Brasil	Pará	São Félix do Xingu	Serra dos Carajás	Arbusto escandente. Folhas discolores. Sépalas verdes; pétalas brancas a

											rosadas com fímbrias vináceas; anteras amarelas; estigmas verdes.
Malpighiaceae	Banisteriopsis	stellaris	(Griseb.) B.Gates	Nogueira, M.G.C.	724	2017	Brasil	Pará	São Félix do Xingu	Serra de Campos	Trepadeira, folhas concolores verdes, pétalas brancas com margem avermelhada.
Malvaceae	Waltheria	indica	L.	Zappi, D.C.	4064	2018	Brasil	Pará	São Félix do Xingu	Serra de Campos, 20 km N de Tancredo Neves	Arbusto esgalhado 1,2 m alt., flores amarelas
Marantaceae	Monotagma	plurispicatum	(Körn.) K.Schum.	Zappi, D.C.	4000	2018	Brasil	Pará	São Félix do Xingu	Serra de Campos, 20 km N de Tancredo Neves	Erva 60 cm alt., inflorescências com brácteas verdes e flores alvas com centro amarelado
Marantaceae	Monotagma	plurispicatum	(Körn.) K.Schum.	Pastore, M.	668	2017	Brasil	Pará	São Félix do Xingu	Serra dos Carajás	Erva ca. 1 m alt. Flores com brácteas castanhas, pedúnculo verde. Estaminódios petalóides brancos.
Marantaceae	Monotagma	plurispicatum	(Körn.) K.Schum.	Andrino, C.O.	628	2019	Brasil	Pará	São Félix do Xingu	Serra de Campos	Erva 1 m., flores passadas castanhas.
Marcgraviaceae	Norantea	guianensis	Aubl.	Zappi, D.C.	3887	2018	Brasil	Pará	São Félix do Xingu	Serra de Campos, 20 km N de Tancredo Neves	Arbusto decumbente, estéril
Melastomataceae	Miconia	alternans	Naudin	Zappi, D.C.	4021	2018	Brasil	Pará	São Félix do Xingu	Serra de Campos, 20 km N de Tancredo Neves	Arbusto aquático 1 m alt., flores alvas
Melastomataceae	Nepsera	aquatica	(Aubl.) Naudim	Andrino, C.O.	649	2019	Brasil	Pará	São Félix do Xingu	Serra de Campos	Cálice verde, Corola alva, anteras lilases.
Melastomataceae	Clidemia	capitellata	(Bonpl.) D.Don	Zappi, D.C.	4020	2018	Brasil	Pará	São Félix do Xingu	Serra de Campos, 20 km N de Tancredo Neves	Arbusto aquático 1,2 m alt., folhas com tricomas rosados, flores cremes, frutos nigrescentes
Melastomataceae	Pleroma	carajasense	K.Rocha, R.Goldenb. & F.S.Mey	Zappi, D.C.	3910	2018	Brasil	Pará	São Félix do Xingu	Serra de Campos, 20 km N de Tancredo Neves	Arbusto 2 m alt., brácteas creme, botões arroxeados
Melastomataceae	Pleroma	carajasense	K.Rocha, R.Goldenb. & F.S.Mey	Pastore, M.	619	2017	Brasil	Pará	São Félix do Xingu	Serra dos Carajás	Arbusto ca. 2 m alt., muito ramificado. Cálice castanho avermelhado; corola branca com tonalidade lilás.
Melastomataceae	Pleroma	carajasense	K.Rocha, R.Goldenb. & F.S.Mey	Nogueira, M.G.C.	708	2017	Brasil	Pará	São Félix do Xingu	Serra de Campos	Arbusto de 1,5 a 1,8 m de alt., ramificado. Folhas discolores, face superior verde mais escuro, cálice verde com lobos castanhos, corola lilás a roxa. Todas plantas em floração.
Melastomataceae	Brasilianthus	carajensis	Almeda & Michelangeli	Zappi, D.C.	3877	2018	Brasil	Pará	São Félix do Xingu	Serra de Campos, 20 km N de	Erva 20 cm, com ramos avermelhados, pétalas rosadas, anteras amarelas

											Tancredo Neves	
Melastomataceae	Brasilianthus		carajensis	Almeda & Michelangeli	Viana, P.L.	6132	0	Brasil	Pará	São Félix do Xingu	Serra de Campos	
Melastomataceae	Brasilianthus		carajensis	Almeda & Michelangeli	Falcão, B.F.	625	2016	Brasil	Pará	São Félix do Xingu	Serra da Seringa	Erva rupícola. Frutos vermelhos. . Pto. E1SER01JUN. DNA ITV2364
Melastomataceae	Brasilianthus		carajensis	Almeda & Michelangeli	Falcão, B.F.	627	2016	Brasil	Pará	São Félix do Xingu	Serra da Seringa	Erva terrestre. Erva pequena formando touceiras com aprox. 13 cm de altura. Folhas com face abaxial avermelhada. Frutos secos. . Pto. E3SER01JUN.
Melastomataceae	Brasilianthus		carajensis	Almeda & Michelangeli	Nogueira, M.G.C.	712	2017	Brasil	Pará	São Félix do Xingu	Serra de Campos	Erva com caule vermelho, muito ramificada, corola lilás.
Melastomataceae	Tibouchina		edmundoi	Brade	Zappi, D.C.	3932	2018	Brasil	Pará	São Félix do Xingu	Serra de Campos, 20 km N de Tancredo Neves	Arbustos ou arvoretas até 3 m alt., frutos passados castanhos
Melastomataceae	Tibouchina		edmundoi	Brade	Falcão, B.F.	631	2016	Brasil	Pará	São Félix do Xingu	Serra da Seringa	Arbustivo. Indivíduo sem folhas, somente com flores da cor roxa.. Pto. E1SER02JUN.
Melastomataceae	Tibouchina		edmundoi	Brade	Falcão, B.F.	651	2016	Brasil	Pará	São Félix do Xingu	Serra da Seringa	Arbusto. Arbusto com flores vistosas róseas.. Pto. E3SER05JUN.
Melastomataceae	Bellucia		grossularioides	(L.) Triana	Zappi, D.C.	3995	2018	Brasil	Pará	São Félix do Xingu	Serra de Campos, 20 km N de Tancredo Neves	Arbusto 4 m alt., estéril
Melastomataceae	Miconia		heliotropoides	Triana	Zappi, D.C.	4008	2018	Brasil	Pará	São Félix do Xingu	Serra de Campos, 20 km N de Tancredo Neves	Arbusto 1 m alt., no subosque, inflorescências castanho-rosadas, flores alvas
Melastomataceae	Miconia		heliotropoides	Triana	Andrino, C.O.	625	2019	Brasil	Pará	São Félix do Xingu	Serra de Campos	Arbusto 1 m, folhas discolores, botões alvos.
Melastomataceae	Miconia		heliotropoides	Triana	Andrino, C.O.	644	2019	Brasil	Pará	São Félix do Xingu	Serra de Campos	Arvore 1,5. Folhas discolores, frutos verdes.
Melastomataceae	Pterolepis		trichotoma	(Rottb.) Cogn.	Zappi, D.C.	4019	2018	Brasil	Pará	São Félix do Xingu	Serra de Campos, 20 km N de Tancredo Neves	Erva 30 cm compr., flores rosadas com anteras amarelas
Menispermaceae	Cissampelos		andromorpha	DC.	Andrino, C.O.	663	2019	Brasil	Pará	São Félix do Xingu	Serra de Campos	Trepadeira. Flores verdes
Menispermaceae	Abuta	cf	grandifolia	(Mart.) Sandwith	Zappi, D.C.	4005	2018	Brasil	Pará	São Félix do Xingu	Serra de Campos, 20 km N de Tancredo Neves	Arbusto semi-escandente, 4 m alt., estéril
Menispermaceae	Abuta		grandifolia	(Mart.) Sandwith	Andrino, C.O.	646	2019	Brasil	Pará	São Félix do Xingu	Serra de Campos	Trepadeira, frutos verdes.
Metteniusaceae	Emmotum		nitens	(Benth.) Miers	Pastore, M.	601	2017	Brasil	Pará	São Félix do Xingu	Serra dos Carajás	Arbusto ca. 2 m alt. Folhas discolores. Frutos verdes.
Myrtaceae	Myrcia		cuprea	(O.Berg.) Kiaersk.	Zappi, D.C.	3933	2018	Brasil	Pará	São Félix do Xingu	Serra de Campos, 20 km N	Arbusto 2,5 m alt., frutos esponjosos vermelhos

										de Tancredo Neves	
Myrtaceae	Myrcia	cuprea	(O.Berg.) Kiaersk.	Zappi, D.C.	3937	2018	Brasil	Pará	São Félix do Xingu	Serra de Campos, 20 km N de Tancredo Neves	Arvoreta 2 m alt., estéril
Myrtaceae	Myrcia	cuprea	(O.Berg.) Kiaersk.	Andrino, C.O.	639	2019	Brasil	Pará	São Félix do Xingu	Serra de Campos	Arbusto 1,5. Folhas com face abaxial dourda brilhante, frutos róseos.
Myrtaceae	Myrciaria	floribunda	(H.West ex Willd.) O.Berg	Zappi, D.C.	3915	2018	Brasil	Pará	São Félix do Xingu	Serra de Campos, 20 km N de Tancredo Neves	Arbusto 1,4 m alt., flores creme, frutos amarelos
Myrtaceae	Myrciaria	floribunda	(H.West ex Willd.) O.Berg	Zappi, D.C.	3992	2018	Brasil	Pará	São Félix do Xingu	Serra de Campos, 20 km N de Tancredo Neves	Arvoreta 3 m alt., flores acastanhadas
Myrtaceae	Myrciaria	glomerata	O.Berg	Zappi, D.C.	4010	2018	Brasil	Pará	São Félix do Xingu	Serra de Campos, 20 km N de Tancredo Neves	Arbusto 4 m alt., estéril
Myrtaceae	Eugenia	punicifolia	(Kunth) DC.	Zappi, D.C.	3894	2018	Brasil	Pará	São Félix do Xingu	Serra de Campos, 20 km N de Tancredo Neves	Arbusto 1.4 m alt., frutos verdes passando a amarelos
Myrtaceae	Eugenia	punicifolia	(Kunth) DC.	Zappi, D.C.	3968	2018	Brasil	Pará	São Félix do Xingu	Serra de Campos, 20 km N de Tancredo Neves	Arvoreta 3 m alt., frutos amarelos passando a vermelhos
Myrtaceae	Myrcia	splendens	(Sw.) DC.	Zappi, D.C.	3965	2018	Brasil	Pará	São Félix do Xingu	Serra de Campos, 20 km N de Tancredo Neves	Arbusto 1 m alt., estéril
Myrtaceae	Myrcia	splendens	(Sw.) DC.	Zappi, D.C.	3994	2018	Brasil	Pará	São Félix do Xingu	Serra de Campos, 20 km N de Tancredo Neves	Árvore 5 m alt., flores alvas
Ochnaceae	Ouratea	castaneifolia	(DC.) Engl.	Zappi, D.C.	3920	2018	Brasil	Pará	São Félix do Xingu	Serra de Campos, 20 km N de Tancredo Neves	Arvoreta 2 m alt., estéril

Ochnaceae	Ouratea		cearensis	(Tiegh.) Sastre & Offroy	Andrino, C.O.	604	2019	Brasil	Pará	São Félix do Xingu	Serra de Campos	Arbusto de 1 m. Calice e corola amarelo. ITV21699
Ochnaceae	Ouratea		racemiformis	Ule	Zappi, D.C.	4033	2018	Brasil	Pará	São Félix do Xingu	Serra de Campos, 20 km N de Tancredo Neves	Arbusto 3 m alt., racemos pendentes esverdeados
Onagraceae	Ludwigia	cf	latifolia	(Benth.) H.Hara	Zappi, D.C.	3967	2018	Brasil	Pará	São Félix do Xingu	Serra de Campos, 20 km N de Tancredo Neves	Subarbusto aquático, estéril
Onagraceae	Ludwigia		nervosa	(Poir.) H.Hara	Andrino, C.O.	674	2019	Brasil	Pará	São Félix do Xingu	Serra de Campos	Arbusto ca. 2 m alt. Flores amarelas. Frutos passados
Orchidaceae	Catasetum		boyi	Mansf.	Silva, J.B.F. da	648	0	Brasil	Pará	São Félix do Xingu		
Orchidaceae	Encyclia		chloroleuca	(Hook.) Neum.	Silva, J.B.F. da	540	0	Brasil	Pará	São Félix do Xingu		
Orchidaceae	Polystachya		concreta	(Jacq.) Garay & H.R.Sweet	Silva, J.B.F. da	225	0	Brasil	Pará	São Félix do Xingu		
Orchidaceae	Polystachya		concreta	(Jacq.) Garay & H.R.Sweet	Andrino, C.O.	669	2019	Brasil	Pará	São Félix do Xingu	Serra de Campos	Erva epífita. Frutos passados.
Orchidaceae	Catasetum		discolor	(Lindl.) Lindl.	Zappi, D.C.	4030	2018	Brasil	Pará	São Félix do Xingu	Serra de Campos, 20 km N de Tancredo Neves	Erva 60 cm alt., flores verdes
Orchidaceae	Catasetum		discolor	(Lindl.) Lindl.	Viana, P.L.	6146	0	Brasil	Pará	São Félix do Xingu	Serra de Campos	
Orchidaceae	Catasetum		discolor	(Lindl.) Lindl.	Pastore, M.	638	2017	Brasil	Pará	São Félix do Xingu	Serra dos Carajás	Erva até 50 cm. Corola castanho-amarelada externamente e castanho-avermelhada internamente.
Orchidaceae	Catasetum		discolor	(Lindl.) Lindl.	Nogueira, M.G.C.	737	2017	Brasil	Pará	São Félix do Xingu	Serra de Campos	Erva terrestre, pseudobulbo verde, flor e frutos verdes.
Orchidaceae	Catasetum		galeritum	Rchb. f.	Silva, J.B.F. da	393	0	Brasil	Pará	São Félix do Xingu		
Orchidaceae	Rodriguezia		lanceolata	Ruiz & Pav.	Andrino, C.O.	665	2019	Brasil	Pará	São Félix do Xingu	Serra de Campos	Erva epífita. Flores roseas
Orchidaceae	Sobralia		liliastrum	Salzm. ex Lindl.	Zappi, D.C.	3888	2018	Brasil	Pará	São Félix do Xingu	Serra de Campos, 20 km N de Tancredo Neves	Erva, estéril
Orchidaceae	Sobralia		liliastrum	Salzm. ex Lindl.	Silva, J.B.F. da	497	0	Brasil	Pará	São Félix do Xingu	Serra de Campo	
Orchidaceae	Sobralia		liliastrum	Salzm. ex Lindl.	Viana, P.L.	6101	0	Brasil	Pará	São Félix do Xingu	Serra de Campos	
Orchidaceae	Sobralia		liliastrum	Salzm. ex Lindl.	Pastore, M.	602	2017	Brasil	Pará	São Félix do Xingu	Serra dos Carajás	Erva ca. 1 m alt. Folhas levemente discolores. Botões verdes amarelados. Cálice verde; corola branca brilhante labelo central amarelo na parte central.
Orchidaceae	Scaphyglottis	cf.	livida	(Lindl.) Schltr.	Andrino, C.O.	671	2019	Brasil	Pará	São Félix do Xingu	Serra de Campos	Erva epífita. Frutos verdes.
Orchidaceae	Rodriguezia		luteola	N.E.Br.	Silva, J.B.F. da	566	0	Brasil	Pará	São Félix do Xingu		

Orchidaceae	Masdevallia	nuda	Lindl	Silva, J.B.F. da	493	0	Brasil	Pará	São Félix do Xingu		
Orchidaceae	Habenaria	nuda	Lindl	Pastore, M.	609	2017	Brasil	Pará	São Félix do Xingu	Serra dos Carajás	Erva ca. 70 cm alt. Folhas concolores; pétalas e sépalas verdes.
Orchidaceae	Habenaria	nuda	Lindl	Nogueira, M.G.C.	749	2017	Brasil	Pará	São Félix do Xingu	Serra de Campos	Erva ereta, cálice e corola verde translúcidos.
Orchidaceae	Habenaria	orchiocalcar	Hoehne	Silva, J.B.F. da	219	0	Brasil	Pará	São Félix do Xingu	Fazenda do Sr. Josué, a 86 Km margem direita do Rio Xingu	
Orchidaceae	Catasetum	planiceps	Lindl.	Zappi, D.C.	3893	2018	Brasil	Pará	São Félix do Xingu	Serra de Campos, 20 km N de Tancredo Neves	Erva crescendo à sombra, pseudobulbo esverdeado, flores passadas alaranjadas
Orchidaceae	Catasetum	planiceps	Lindl.	Silva, J.B.F. da	3219	0	Brasil	Pará	São Félix do Xingu	Serra de Campo	
Orchidaceae	Erycina	pusilla	(L.) N.H.Williams & M.W.Chase	Silva, J.B.F. da	498	0	Brasil	Pará	São Félix do Xingu		
Orchidaceae	Erycina	pusilla	(L.) N.H.Williams & M.W.Chase	Nogueira, M.G.C.	707	2017	Brasil	Pará	São Félix do Xingu	Serra de Campos	Epífita mínima, raízes enroladas sobre a planta. Folhas patentes. Face superior ligeiramente verde mais escuro, flores amarelas.
Orchidaceae	Epidendrum	strobiliferum	Rchb.f.	Andrino, C.O.	667	2019	Brasil	Pará	São Félix do Xingu	Serra de Campos	Erva epífita. Frutos verdes.
Orobanchaceae	Buchnera	carajasensis	Scatigna & N.Mota	Zappi, D.C.	3931	2018	Brasil	Pará	São Félix do Xingu	Serra de Campos, 20 km N de Tancredo Neves	Erva com caules quebradiços, atingindo 40 cm alt., flores rosa-arroxeadas com a fauce alva
Orobanchaceae	Buchnera	carajasensis	Scatigna & N.Mota	Viana, P.L.	6135	2016	Brasil	Pará	São Félix do Xingu	Serra de Campos	
Orobanchaceae	Buchnera	carajasensis	Scatigna & N.Mota	Falcão, B.F.	626	2016	Brasil	Pará	São Félix do Xingu	Serra da Seringa	Erva rupícola. Corola lilás. Fruto negro. Pto. E1SER03JUN. DNA ITV2371
Orobanchaceae	Buchnera	carajasensis	Scatigna & N.Mota	Pastore, M.	610	2017	Brasil	Pará	São Félix do Xingu	Serra dos Carajás	Erva ca. 50 cm alt. Folhas concolores. Cálice verde a vináceo; corola lilás com o tubo mais escuro. Frutos verdes. ITV 1953
Orobanchaceae	Buchnera	carajasensis	Scatigna & N.Mota	Pastore, M.	646	2017	Brasil	Pará	São Félix do Xingu	Serra dos Carajás	Erva ca. 30 cm alt. Cálice verde, flores com corola roxa e flores com corola branca. ITV 1962.
Passifloraceae	Passiflora	ceratocarpa	F. Silveira	Zappi, D.C.	4060	2018	Brasil	Pará	São Félix do Xingu	Serra de Campos, 20 km N de Tancredo Neves	Trepadeira robusta, fruto amarelo
Passifloraceae	Passiflora	ceratocarpa	F. Silveira	Andrino, C.O.	620	2019	Brasil	Pará	São Félix do Xingu	Serra de Campos	Trepadeira, Cálice alvo com nervura central verde, corola alva. ITV21706.
Passifloraceae	Passiflora	picturata	Ker Gawl.	Zappi, D.C.	3976	2018	Brasil	Pará	São Félix do Xingu	Serra de Campos, 20 km N de	Trepadeira, flores roxas com corona alva e roxa

											Tancredo Neves	
Passifloraceae	Passiflora		tholozanii	Sacco	Andrino, C.O.	612	2019	Brasil	Pará	São Félix do Xingu	Serra de Campos	Cálice verde alaranjado e corola vermelha.
Phyllanthaceae	Phyllanthus		hyssopifolioides	Kunth.	Zappi, D.C.	4028	2018	Brasil	Pará	São Félix do Xingu	Serra de Campos, 20 km N de Tancredo Neves	Erva aquática, caules avermelhados, frutos verdes
Phyllanthaceae	Phyllanthus		minutulus	Müll.Arg.	Zappi, D.C.	4026	2018	Brasil	Pará	São Félix do Xingu	Serra de Campos, 20 km N de Tancredo Neves	Erva em solo encharcado, ramos avermelhados, flores creme, frutos verdes
Phytolaccaceae	Phytolacca		thyrsiflora	Fenzl ex J. Schmidt	Zappi, D.C.	4041	2018	Brasil	Pará	São Félix do Xingu	Serra de Campos, 20 km N de Tancredo Neves	Erva 1,4 m alt., inflorescências rosadas, frutos arroxeados
Piperaceae	Peperomia		albopilosa	D. Monteiro	Viana, P.L.	6169	2016	Brasil	Pará	São Félix do Xingu	Serra de Campos	Erva anual, rupícola em interior de mata baixa em drenagem. Folhas discolores.
Piperaceae	Peperomia		magnoliifolia	(Jacq.) A.Dietr.	Andrino, C.O.	647	2019	Brasil	Pará	São Félix do Xingu	Serra de Campos	Epífita. Espigas creme.
Plantaginaceae	Scoparia		dulcis	L.	Zappi, D.C.	4065	2018	Brasil	Pará	São Félix do Xingu	Serra de Campos, 20 km N de Tancredo Neves	Erva 20 cm alt., flores alvas
Poaceae	Trichantecium		arctum	(Swallen) Zuloaga & Morrone	Zappi, D.C.	3913	2018	Brasil	Pará	São Félix do Xingu	Serra de Campos, 20 km N de Tancredo Neves	Erva com frutos verde-claros
Poaceae	Trichanthecium	cf	arctum	(Swallen) Zuloaga & Morrone	Viana, P.L.	6133	0	Brasil	Pará	São Félix do Xingu	Serra de Campos	
Poaceae	Trichantecium	cf	arctum	(Swallen) Zuloaga & Morrone	Pastore, M.	604	2017	Brasil	Pará	São Félix do Xingu	Serra dos Carajás	Erva em canga alagada. Folhas concolores. Espiguetas verdes.
Poaceae	Trichanthecium	cf	arctum	(Swallen) Zuloaga & Morrone	Pastore, M.	628	2017	Brasil	Pará	São Félix do Xingu	Serra dos Carajás	Erva associada à lateral da rocha.
Poaceae	Paspalum		axillare	Swallen	Viana, P.L.	6130	0	Brasil	Pará	São Félix do Xingu	Serra de Campos	
Poaceae	Paspalum		axillare	Swallen	Nogueira, M.G.C.	727	2017	Brasil	Pará	São Félix do Xingu	Serra de Campos	Erva com inflorescência verde.
Poaceae	Andropogon		bicornis	L.	Zappi, D.C.	3950	2018	Brasil	Pará	São Félix do Xingu	Serra de Campos, 20 km N de Tancredo Neves	Erva 1,5 m alt., inflorescências castanhas com tricomas alvos
Poaceae	Ichnanthus		calvescens	(Nees ex Trin.) Döll	Zappi, D.C.	4011	2018	Brasil	Pará	São Félix do Xingu	Serra de Campos,	Erva 1,8 m alt., inflorescências verde-claras

											20 km N de Tancredo Neves	
Poaceae	Mesosetum		cayennense	Steud.	Viana, P.L.	6117	0	Brasil	Pará	São Félix do Xingu	Serra de Campos	
Poaceae	Paspalum		foliiforme	S.Denham	Zappi, D.C.	3916	2018	Brasil	Pará	São Félix do Xingu	Serra de Campos, 20 km N de Tancredo Neves	Erva 40 cm alt., espigas esverdeadas, anteras alvas
Poaceae	Paspalum		foliiforme	S.Denham	Zappi, D.C.	4009	2018	Brasil	Pará	São Félix do Xingu	Serra de Campos, 20 km N de Tancredo Neves	Erva em touceiras, 60 cm alt., inflorescências verde-claras
Poaceae	Paspalum	cf	foliiforme	S. Denham	Viana, P.L.	6165	0	Brasil	Pará	São Félix do Xingu	Serra de Campos	
Poaceae	Paspalum		foliiforme	S. Denham	Viana, P.L.	6128	0	Brasil	Pará	São Félix do Xingu	Serra de Campos	
Poaceae	Oryza		glumaepatula	Steud.	Falcão, B.F.	634	2016	Brasil	Pará	São Félix do Xingu	Serra da Seringa	Erva aquática. Gramínea com ca. de 45 cm de altura semelhante ao arroz. Frutos amarelos esverdeados. . Pto. E3SER02JUN. DNA ITV2375
Poaceae	Oryza		glumaepatula	Steud.	Falcão, B.F.	645	2016	Brasil	Pará	São Félix do Xingu	Serra da Seringa	Erva aquática emergente. Inflorescência esverdeada com anteras brancas. Pto. E1SER08JUN. DNA ITV2392 e ITV2393
Poaceae	Oryza		glumaepatula	Steud.	Pastore, M.	626	2017	Brasil	Pará	São Félix do Xingu	Serra dos Carajás	Erva até 80 cm alt. Espiguetas verdes tornando-se paleácea. ITV 1968.
Poaceae	Rhytachne		gonzalezii	Davidse	Viana, P.L.	6127	0	Brasil	Pará	São Félix do Xingu	Serra de Campos	
Poaceae	Steinchisma		laxum	(Sw.) Zuloaga	Andrino, C.O.	677	2019	Brasil	Pará	São Félix do Xingu	Serra de Campos	Erva rupícola. Inflorescência verde
Poaceae	Axonopus	cf	longispicus	(Döll) Kuhlm.	Zappi, D.C.	4023	2018	Brasil	Pará	São Félix do Xingu	Serra de Campos, 20 km N de Tancredo Neves	Erva 30 cm alt., inflorescências creme, em solo encharcado
Poaceae	Urochloa		maxima	(Jacq.) R. Webster	Zappi, D.C.	3951	2018	Brasil	Pará	São Félix do Xingu	Serra de Campos, 20 km N de Tancredo Neves	Erva 2 m alt., inflorescências verdes
Poaceae	Parodiolyra		micrantha	(Kunth) Davidse & Zuloaga	Zappi, D.C.	3999	2018	Brasil	Pará	São Félix do Xingu	Serra de Campos, 20 km N de Tancredo Neves	Bambu crescendo à sombra, 2 m alt.
Poaceae	Melinis		minutiflora	P.Beauv.	Zappi, D.C.	3972	2018	Brasil	Pará	São Félix do Xingu	Serra de Campos, 20 km N de Tancredo Neves	Erva na beira da estrada, estéril

Poaceae	Melinis	minutiflora	P.Beauv.	Andrino, C.O.	640	2019	Brasil	Pará	São Félix do Xingu	Serra de Campos	Erva, Glumas vináceas, anteras amarelas.
Poaceae	Hildaea	parvispiculata	C. Silva & R.P. Oliveira	Viana, P.L.	6124	0	Brasil	Pará	São Félix do Xingu	Serra de Campos	
Poaceae	Luziola	peruviana	Juss. ex J.F.Gmel.	Zappi, D.C.	3918	2018	Brasil	Pará	São Félix do Xingu	Serra de Campos, 20 km N de Tancredo Neves	Erva aquática, gluma lilás, anteras creme
Poaceae	Luziola	peruviana	Juss. ex J.F.Gmel.	Viana, P.L.	6156	0	Brasil	Pará	São Félix do Xingu	Serra de Campos	
Poaceae	Luziola	peruviana	Juss. ex J.F.Gmel.	Pastore, M.	605	2017	Brasil	Pará	São Félix do Xingu	Serra dos Carajás	Erva aquática. Espiguetas creme; anteras rosadas.
Poaceae	Rugoloa	pilosa	(Sw.) Zuloaga	Zappi, D.C.	3964	2018	Brasil	Pará	São Félix do Xingu	Serra de Campos, 20 km N de Tancredo Neves	Erva aquática, inflorescências verdes
Poaceae	Paspalum	reticulinerve	Renvoize	Viana, P.L.	6166	0	Brasil	Pará	São Félix do Xingu	Serra de Campos	
Poaceae	Paspalum	reticulinerve	Renvoize	Falcão, B.F.	648	2016	Brasil	Pará	São Félix do Xingu	Serra da Seringa	Erva terrestre. Frutos esbranquiçados dispersando. Pto. E1SER11JUN.
Poaceae	Paspalum	reticulinerve	Renvoize	Pastore, M.	640	2017	Brasil	Pará	São Félix do Xingu	Serra dos Carajás	Erva com inflorescências vináceas.
Poaceae	Paspalum	reticulinerve	Renvoize	Pastore, M.	653	2017	Brasil	Pará	São Félix do Xingu	Serra dos Carajás	Erva ereta. Folhas verdes concolores. Parte externa da raque da inflorescência verde e a externa roxa a negrescente.
Poaceae	Paspalum	reticulinerve	Renvoize	Nogueira, M.G.C.	729	2017	Brasil	Pará	São Félix do Xingu	Serra de Campos	Erva sobre canga coberta de briófitas, folhas verdes, espiguetas vináceas a negras.
Poaceae	Axonopus	rupestris	Davidse	Zappi, D.C.	3896	2018	Brasil	Pará	São Félix do Xingu	Serra de Campos, 20 km N de Tancredo Neves	Touceiras com inflorescências em V, anteras arroxeadas
Poaceae	Trachypogon	spicatus	(L.f.) Kuntze	Zappi, D.C.	3944	2018	Brasil	Pará	São Félix do Xingu	Serra de Campos, 20 km N de Tancredo Neves	Erva em touceiras 1,2 m alt., inflorescências avermelhadas
Poaceae	Trachypogon	spicatus	(L.f.) Kuntze	Pastore, M.	654	2017	Brasil	Pará	São Félix do Xingu	Serra dos Carajás	Erva ereta, cespitosa. Folhas verdes a paleáceas. Espiguetas verdes a vináceaes com tricomas brancos.
Poaceae	Acroceras	zizanioides	(Kunth) Dandy	Zappi, D.C.	4022	2018	Brasil	Pará	São Félix do Xingu	Serra de Campos, 20 km N de Tancredo Neves	Erva aquática, frutos verde-claros
Polygalaceae	Polygala	adenophora	DC.	Zappi, D.C.	3900	2018	Brasil	Pará	São Félix do Xingu	Serra de Campos, 20 km N de Tancredo Neves	Erva 10-15 cm alt., flores rosa-magenta

Polygalaceae	Polygala	adenophora	DC.	Pastore, M.	645	2017	Brasil	Pará	São Félix do Xingu	Serra dos Carajás	Erva. Cálice e corola lilás.
Polygalaceae	Polygala	adenophora	DC.	Nogueira, M.G.C.	711	2017	Brasil	Pará	São Félix do Xingu	Serra de Campos	Erva, cerca de 30 cm de altura, flores roxas, pétala interna com mancha branca.
Polygalaceae	Bredemeyera	divaricata	(DC.) J.F.B. Pastore	Zappi, D.C.	3911	2018	Brasil	Pará	São Félix do Xingu	Serra de Campos, 20 km N de Tancredo Neves	Arbusto 1,6 m alt., pétalas alvas
Polygalaceae	Bredemeyera	divaricata	(DC.) J.F.B. Pastore	Nogueira, M.G.C.	720	2017	Brasil	Pará	São Félix do Xingu	Serra de Campos	Arbusto com cerca de 2 m de alt., sépalas creme e internas com mancha amarelada.
Polygalaceae	Caamembeca	spectabilis	(DC.) J.F.B. Pastore	Zappi, D.C.	3908	2018	Brasil	Pará	São Félix do Xingu	Serra de Campos, 20 km N de Tancredo Neves	Subarbutsto esgalhado 1 m alt., pétalas externas rosadas, internas creme, apicalmente amarelas
Polygalaceae	Caamembeca	spectabilis	(DC.) J.F.B. Pastore	Pastore, M.	632	2017	Brasil	Pará	São Félix do Xingu	Serra dos Carajás	Arbusto até 1 m alt. Folhas vináceas na face adaxial e verdes na face abaxial com nervuras vináceas. Sépalas verdes e pétalas verdes.
Polygalaceae	Caamembeca	spectabilis	(DC.) J.F.B. Pastore	Nogueira, M.G.C.	718	2017	Brasil	Pará	São Félix do Xingu	Serra de Campos	Erva com cerca de 50 cm de altura, sépalas externas rosadas e interna amareladas.
Polygalaceae	Caamembeca	spectabilis	(DC.) J.F.B. Pastore	Andrino, C.O.	642	2019	Brasil	Pará	São Félix do Xingu	Serra de Campos	Arbusto 80 cm, botões vináceos.
Polypodiaceae	Microgramma	persicariifolia	(Schrad.) C.Presl	Zappi, D.C.	4066	2018	Brasil	Pará	São Félix do Xingu	Serra de Campos, 20 km N de Tancredo Neves	Samambaia crescendo sobre ramos, soros castanhos
Polypodiaceae	Pleopeltis	polypodioides	(L.) Andrews & Windham	Zappi, D.C.	3922	2018	Brasil	Pará	São Félix do Xingu	Serra de Campos, 20 km N de Tancredo Neves	Samambaia rasteira crescendo à sombra, soros ferrugíneos
Polypodiaceae	Serpocaulon	triseriale	(Sw.) A.R.Sm.	Zappi, D.C.	4037	2018	Brasil	Pará	São Félix do Xingu	Serra de Campos, 20 km N de Tancredo Neves	Samambaia crescendo à sombra, estéril
Portulacaceae	Portulaca	sedifolia	N.E.Br.	Zappi, D.C.	3862	2018	Brasil	Pará	São Félix do Xingu	Serra de Campos, 20 km N de Tancredo Neves	Erva com folhas suculentas verde amareladas, flores alvas
Portulacaceae	Portulaca	sedifolia	N.E.Br.	Viana, P.L.	6131	0	Brasil	Pará	São Félix do Xingu	Serra de Campos	
Portulacaceae	Portulaca	sedifolia	N.E.Br.	Viana, P.L.	6177	0	Brasil	Pará	São Félix do Xingu	Serra de Campos	
Portulacaceae	Portulaca	sedifolia	N.E.Br.	Falcão, B.F.	633	2016	Brasil	Pará	São Félix do Xingu	Serra da Seringa	Herbácea. Pilosa. Frutos secos bege. Pto. E2SER01JUN.

Portulacaceae	Portulaca	sedifolia	N.E.Br.	Pastore, M.	670	2017	Brasil	Pará	São Félix do Xingu	Serra dos Carajás	Erva com ramos eretos e prostrados, com indumento branco, muito denso. Folhas avermelhadas a verdes com ornamentação verde. Corola branca. ITV 1957.
Primulaceae	Cybianthus	detergens	Mart.	Zappi, D.C.	4062	2018	Brasil	Pará	São Félix do Xingu	Serra de Campos, 20 km N de Tancredo Neves	Arbusto 3 m alt., inflorescências amareladas
Proteaceae	Roupala	montana	Aubl.	Zappi, D.C.	4063	2018	Brasil	Pará	São Félix do Xingu	Serra de Campos, 20 km N de Tancredo Neves	Arbusto 2 m alt., folhas jovens pinadas, maduras inteiras, estéril
Pteridaceae	Doryopteris	collina	(Raddi) J.Sm.	Zappi, D.C.	4040	2018	Brasil	Pará	São Félix do Xingu	Serra de Campos, 20 km N de Tancredo Neves	Samambaia crescendo entre pedras, ao sol, soros castanhos
Rhamnaceae	Gouania	pyrifolia	Reissek	Zappi, D.C.	3953	2018	Brasil	Pará	São Félix do Xingu	Serra de Campos, 20 km N de Tancredo Neves	Trepadeira estéril
Rhamnaceae	Gouania	pyrifolia	Reissek	Viana, P.L.	6172	0	Brasil	Pará	São Félix do Xingu	Serra de Campos	
Rubiaceae	Borreria	alata	(Aubl.) DC.	Zappi, D.C.	3866	2018	Brasil	Pará	São Félix do Xingu	Serra de Campos, 20 km N de Tancredo Neves	Erva crescendo à sombra, flores alvas
Rubiaceae	Guettarda	argentea	Lam.	Andrino, C.O.	602	2019	Brasil	Pará	São Félix do Xingu	Serra de Campos	Arbusto muito ramificado até 3 m. Flores creme. ITV21700
Rubiaceae	Borreria	carajasensis	E.L. Cabral & L.M. Miguel	Zappi, D.C.	3859	2018	Brasil	Pará	São Félix do Xingu	Serra de Campos, 20 km N de Tancredo Neves	Subarbusto 40 cm alt., com ramos avermelhados, flores alvas
Rubiaceae	Borreria	carajasensis	E.L. Cabral & L.M. Miguel	Zappi, D.C.	3906	2018	Brasil	Pará	São Félix do Xingu	Serra de Campos, 20 km N de Tancredo Neves	Erva 15-20 cm alt., flores alvas
Rubiaceae	Borreria	carajasensis	E.L. Cabral & L.M. Miguel	Zappi, D.C.	3926	2018	Brasil	Pará	São Félix do Xingu	Serra de Campos, 20 km N de Tancredo Neves	Subarbusto 30 cm alt., flores alvas
Rubiaceae	Borreria	carajasensis	E.L. Cabral & L.M. Miguel	Viana, P.L.	6112	0	Brasil	Pará	São Félix do Xingu	Serra de Campos	

Rubiaceae	Borreria	carajasensis	E.L. Cabral & L.M. Miguel	Falcão, B.F.	615	2016	Brasil	Pará	São Félix do Xingu	Serra da Seringa	Erva rupícola. Frutos bege esverdeados. Pto. E1SER01JUN. DNA ITV2381
Rubiaceae	Borreria	carajasensis	E.L. Cabral & L.M. Miguel	Falcão, B.F.	620	2016	Brasil	Pará	São Félix do Xingu	Serra da Seringa	Subarbusto. Corola branca. Frutos verde-claro. Pto. E2SER01JUN.
Rubiaceae	Borreria	carajasensis	E.L. Cabral & L.M. Miguel	Falcão, B.F.	639	2016	Brasil	Pará	São Félix do Xingu	Serra da Seringa	Erva rupícola. Corola alva. Frutos verde-claro. Pto. E1SER08JUN. DNA ITV2389
Rubiaceae	Borreria	carajasensis	E.L. Cabral & L.M. Miguel	Falcão, B.F.	641	2016	Brasil	Pará	São Félix do Xingu	Serra da Seringa	Erva terrestre. Frutos escuros, dispersando.. Pto. E1SER11JUN.
Rubiaceae	Borreria	carajasensis	E.L. Cabral & L.M. Miguel	Pastore, M.	658	2017	Brasil	Pará	São Félix do Xingu	Serra dos Carajás	Erva delicada. Cálice verde e corola branca; anteras e estigma brancos. ITV 1956.
Rubiaceae	Borreria	carajasensis	E.L. Cabral & L.M. Miguel	Nogueira, M.G.C.	704	2017	Brasil	Pará	São Félix do Xingu	Serra de Campos	Erva com cerca de 30 cm, muito ramificada, ramos prostrados, eretos e avermelhados, Estípulas avermelhadas, brácteas verdes, corola, antera e estilete brancos.
Rubiaceae	Borreria	carajasensis	E.L. Cabral & L.M. Miguel	Andrino, C.O.	636	2019	Brasil	Pará	São Félix do Xingu	Serra de Campos	Flores com cálice verde e corola alva.
Rubiaceae	Perama	carajensis	J.H. Kirkbr.	Zappi, D.C.	3879	2018	Brasil	Pará	São Félix do Xingu	Serra de Campos, 20 km N de Tancredo Neves	Erva 40 cm alt., folhas perfoliadas armazenando água, flores azul-claras
Rubiaceae	Perama	carajensis	J.H. Kirkbr.	Viana, P.L.	6103	0	Brasil	Pará	São Félix do Xingu	Serra de Campos	
Rubiaceae	Perama	carajensis	J.H.Kirkbr.	Nogueira, M.G.C.	713	2017	Brasil	Pará	São Félix do Xingu	Serra de Campos	Erva com cerca de 30 a 60 cm de altura, folha verdes a vermelhas.
Rubiaceae	Psychotria	colorata	(Willd. ex Schult.) Mull. Arg.	Zappi, D.C.	4017	2018	Brasil	Pará	São Félix do Xingu	Serra de Campos, 20 km N de Tancredo Neves	Erva 30 cm alt., brácteas avermelhadas, frutos azuis
Rubiaceae	Alibertia	edulis	(Rich.) A. Rich. ex DC.	Zappi, D.C.	4035	2018	Brasil	Pará	São Félix do Xingu	Serra de Campos, 20 km N de Tancredo Neves	Arbusto pouco ramificado, estéril
Rubiaceae	Sabicea	grisea	Cham. & Schltdl.	Zappi, D.C.	3901	2018	Brasil	Pará	São Félix do Xingu	Serra de Campos, 20 km N de Tancredo Neves	Trepadeira, frutos rosados
Rubiaceae	Palicourea	guianensis	Aubl.	Zappi, D.C.	4052	2018	Brasil	Pará	São Félix do Xingu	Serra de Campos, 20 km N de Tancredo Neves	Arvoreta 5 m alt., no interior do capão, estéril
Rubiaceae	Coutarea	hexandra	(Jacq.) K.Schum.	Andrino, C.O.	610	2019	Brasil	Pará	São Félix do Xingu	Serra de Campos	Arbusto 1,5 m. Frutos secos castanhos. ITV21701
Rubiaceae	Psychotria	hoffmannseggiana	(Willd. ex Schult.) Mull. Arg.	Zappi, D.C.	3939	2018	Brasil	Pará	São Félix do Xingu	Serra de Campos, 20 km N de	Subarbusto 40 cm alt., estéril

											Tancredo Neves	
Rubiaceae	Psychotria	cf	hoffmannseggiana	(Willd. ex Schult.) Mull. Arg.	Zappi, D.C.	3989	2018	Brasil	Pará	São Félix do Xingu	Serra de Campos, 20 km N de Tancredo Neves	Subarbustos 1,2 m alt., frutos verdes, listrados
Rubiaceae	Psychotria		hoffmannseggiana	(Willd. ex Schult.) Mull. Arg.	Andrino, C.O.	601	2019	Brasil	Pará	São Félix do Xingu	Serra de Campos	Arbusto 70 cm., folhas discolores, inflorescencia creme esverdeada.
Rubiaceae	Cordiera		myrciifolia	(K.Schum.) C.H.Perss. & Delprete	Zappi, D.C.	3971	2018	Brasil	Pará	São Félix do Xingu	Serra de Campos, 20 km N de Tancredo Neves	Arbusto 1 m alt., estéril
Rubiaceae	Borreria		semiamplexicaulis	E.L.Cabral	Zappi, D.C.	3938	2018	Brasil	Pará	São Félix do Xingu	Serra de Campos, 20 km N de Tancredo Neves	Erva crescendo à sombra, 40 cm alt., flores alvas
Rutaceae	Pilocarpus		microphyllus	Stapf ex Wardlew.	Zappi, D.C.	3978	2018	Brasil	Pará	São Félix do Xingu	Serra de Campos, 20 km N de Tancredo Neves	Arbusto no subosque, 1,5 m alt., inflorescências verdes, flores amareladas
Rutaceae	Pilocarpus		microphyllus	Stapf ex Wardlew.	Falcão, B.F.	638	2016	Brasil	Pará	São Félix do Xingu	Serra da Seringa	Arbusto terrícola. Arbusto ca. 1,5m de altura. Frutos verdes. Pto. E3SER06JUN. DNA ITV2374
Rutaceae	Pilocarpus		microphyllus	Stapf ex Wardlew.	Nogueira, M.G.C.	744	2017	Brasil	Pará	São Félix do Xingu	Serra de Campos	Arbusto até 1,5 m de altura, decumbente com ramos pendentes, folíolos discolores com glândulas.
Rutaceae	Pilocarpus		microphyllus	Stapf ex Wardlew.	Andrino, C.O.	653	2019	Brasil	Pará	São Félix do Xingu	Serra de Campos	Frutos verdes.
Rutaceae	Ertela		trifolia	(L.) Kuntze	Zappi, D.C.	3956	2018	Brasil	Pará	São Félix do Xingu	Serra de Campos, 20 km N de Tancredo Neves	Subarbusto 40 cm alt., flores alvas
Rutaceae	Ertela		trifolia	(L.) Kuntze	Nogueira, M.G.C.	714	2017	Brasil	Pará	São Félix do Xingu	Serra de Campos	Subarbusto com cerca de 30 cm de altura, caule esbranquiçado, folhas verdes com tom amarelado, concolores, pendentes, brácteas verdes e pétalas brancas.
Rutaceae	Ertela		trifolia	(L.) Kuntze	Andrino, C.O.	607	2019	Brasil	Pará	São Félix do Xingu	Serra de Campos	Arbusto 60 cm. Flores alvas.
Rutaceae	Dictyoloma		vandellianum	A. Juss.	Zappi, D.C.	3975	2018	Brasil	Pará	São Félix do Xingu	Serra de Campos, 20 km N de Tancredo Neves	Árvoretas 5 m alt., flores creme
Rutaceae	Dictyoloma		vandellianum	A. Juss.	Viana, P.L.	6148	0	Brasil	Pará	São Félix do Xingu	Serra de Campos	
Rutaceae	Dictyoloma		vandellianum	A. Juss.	Nogueira, M.G.C.	733	2017	Brasil	Pará	São Félix do Xingu	Serra de Campos	Árvore com cerca de 5 m, dap cerca de 96 cm, tronco branco, folhas

												avermelhadas no ápice, discolores, face superior verde-escuro e inferior verde amarelado, frutos passados.
Salicaceae	Casearia		arborea	(Rich.) Urb.	Zappi, D.C.	3982	2018	Brasil	Pará	São Félix do Xingu	Serra de Campos, 20 km N de Tancredo Neves	Árvore 5 m alt., flores creme, frutos atropurpúreos
Salicaceae	Casearia		grandiflora	Cambess,	Andrino, C.O.	606	2019	Brasil	Pará	São Félix do Xingu	Serra de Campos	Árvore. Fruto castanho escuro, semente com arilo laranja
Salicaceae	Casearia		javitensis	Kunth	Zappi, D.C.	4014	2018	Brasil	Pará	São Félix do Xingu	Serra de Campos, 20 km N de Tancredo Neves	Arbusto 4 m alt., estéril
Salicaceae	Casearia		javitensis	Kunth	Andrino, C.O.	656	2019	Brasil	Pará	São Félix do Xingu	Serra de Campos	Árvore 5 m. Frutos vínaceos.
Sapindaceae	Allophylus		edulis	(A.St.-Hil. et al.) Hieron. ex Niederl.	Zappi, D.C.	3959	2018	Brasil	Pará	São Félix do Xingu	Serra de Campos, 20 km N de Tancredo Neves	Arbusto 1,5 m alt., frutos amarelos
Sapindaceae	Pseudima		frutescens	(Aubl.) Radlk.	Viana, P.L.	6151	0	Brasil	Pará	São Félix do Xingu	Serra de Campos	
Sapindaceae	Serjania		lethalis	A.St.-Hil.	Zappi, D.C.	3996	2018	Brasil	Pará	São Félix do Xingu	Serra de Campos, 20 km N de Tancredo Neves	Trepadeira estéril
Sapindaceae	Paullinia		stellata	Radlk.	Zappi, D.C.	4044	2018	Brasil	Pará	São Félix do Xingu	Serra de Campos, 20 km N de Tancredo Neves	Trepadeira na beira do capão de mata, frutos vermelho-vivo
Sapotaceae	Pouteria		ramiflora	(Mart.) Radlk.	Zappi, D.C.	3969	2018	Brasil	Pará	São Félix do Xingu	Serra de Campos, 20 km N de Tancredo Neves	Arvoreta 3 m alt., frutos velhos secos
Sapotaceae	Pouteria	cf	ramiflora	(Mart.) Radlk.	Zappi, D.C.	3979	2018	Brasil	Pará	São Félix do Xingu	Serra de Campos, 20 km N de Tancredo Neves	Árvore 3 m alt., frutos verdes
Sapotaceae	Pouteria		ramiflora	(Mart.) Radlk.	Andrino, C.O.	634	2019	Brasil	Pará	São Félix do Xingu	Serra de Campos	Arbusto 2 m. Frutos verdes.
Sellaginellaceae	Selaginella		radiata	(Aubl.) Spring.	Zappi, D.C.	4055	2018	Brasil	Pará	São Félix do Xingu	Serra de Campos, 20 km N de Tancredo Neves	Samambaia crescendo à sombra no interior do capão, estéril

Simaroubaceae	Simarouba	amara	Aubl.	Zappi, D.C.	3985	2018	Brasil	Pará	São Félix do Xingu	Serra de Campos, 20 km N de Tancredo Neves	Arbusto na beira da canga, estéril
Simaroubaceae	Simaba	guianensis	Aubl.	Zappi, D.C.	3984	2018	Brasil	Pará	São Félix do Xingu	Serra de Campos, 20 km N de Tancredo Neves	Arbusto na beira da canga, estéril
Siparunaceae	Siparuna	ficoides	S.S.Rener & Hausner	Andrino, C.O.	660	2019	Brasil	Pará	São Félix do Xingu	Serra de Campos	Ávore 2 m. Odor desagradável.
Smilacaceae	Smilax	irrorata	Mart. ex Griseb	Zappi, D.C.	3935	2018	Brasil	Pará	São Félix do Xingu	Serra de Campos, 20 km N de Tancredo Neves	Trepadeira com frutos verdes passando a enegrecidos
Smilacaceae	Smilax	irrorata	Mart. ex Griseb	Zappi, D.C.	4039	2018	Brasil	Pará	São Félix do Xingu	Serra de Campos, 20 km N de Tancredo Neves	Trepadeira crescendo sobre arbustos, estéril
Smilacaceae	Smilax	irrorata	Mart. ex Griseb	Viana, P.L.	6122	0	Brasil	Pará	São Félix do Xingu	Serra de Campos	
Smilacaceae	Smilax	irrorata	Mart. ex Griseb	Pastore, M.	669	2017	Brasil	Pará	São Félix do Xingu	Serra dos Carajás	Trepadeira com gavinhas. Folhas discolores. Frutos verdes, roxos ou pretos.
Smilacaceae	Smilax	irrorata	Mart. ex Griseb	Nogueira, M.G.C.	702	2017	Brasil	Pará	São Félix do Xingu	Serra de Campos	Subarbusto sobre canga, 30 cm de altura, botões verdes.
Smilacaceae	Smilax	irrorata	Mart. ex Griseb	Nogueira, M.G.C.	732	2017	Brasil	Pará	São Félix do Xingu	Serra de Campos	Trepadeira, folhas verde-escuras, frutos verdes.
Smilacaceae	Smilax	irrorata	Mart. ex Griseb	Andrino, C.O.	626	2019	Brasil	Pará	São Félix do Xingu	Serra de Campos	Trepadeira, frutos jovens verdes.
Smilacaceae	Smilax	irrorata	Mart. ex Griseb	Andrino, C.O.	627	2019	Brasil	Pará	São Félix do Xingu	Serra de Campos	Trepadeira, flores verdes.
Solanaceae	Solanum	americanum	Mill.	Zappi, D.C.	4059	2018	Brasil	Pará	São Félix do Xingu	Serra de Campos, 20 km N de Tancredo Neves	Erva 30 cm alt., crescendo à sombra, flores alvas com estames amarelos, frutos verdes
Solanaceae	Solanum	crinitum	Lam.	Andrino, C.O.	623	2019	Brasil	Pará	São Félix do Xingu	Serra de Campos	Arvoreta 4 m. Frutos verdes
Trigoniaceae	Trigonia	nivea	Cambess.	Zappi, D.C.	3952	2018	Brasil	Pará	São Félix do Xingu	Serra de Campos, 20 km N de Tancredo Neves	Trepadeira, flores alvas com centro rosa e amarelo
Trigoniaceae	Trigonia	nivea	Cambess.	Andrino, C.O.	651	2019	Brasil	Pará	São Félix do Xingu	Serra de Campos	Arbusto escandente, 1 m. Folhas discolores, flor creme internamente alva com anteras amarelas.
Turneraceae	Turnera	glaziovii	Urb	Zappi, D.C.	4012	2018	Brasil	Pará	São Félix do Xingu	Serra de Campos, 20 km N de	Arbusto 1 m alt., estéril

													Tancredo Neves	
Turneraceae	Turnera	laciniata	Arbo				Zappi, D.C.	3993	2018	Brasil	Pará	São Félix do Xingu	Serra de Campos, 20 km N de Tancredo Neves	Erva crescendo em locais sombreados, 50 cm alt., flores laranja, frutos verde-claros
Turneraceae	Turnera	laciniata	Arbo				Nogueira, M.G.C.	741	2017	Brasil	Pará	São Félix do Xingu	Serra de Campos	Arbusto com cerca de 2 m de altura, lâminas discolores, sépalas verdes, cálice laranja. Habitando a margem da floresta, área sombreada.
Turneraceae	Turnera	melochioides	Cambess.	var.	latifolia	Urb.	Viana, P.L.	6160	2016	Brasil	Pará	São Félix do Xingu	Serra de Campos	Arbusto 0,5 m. de altura com flores amarelas.
Turneraceae	Turnera	melochioides	Cambess.	var.	latifolia	Urb.	Pastore, M.	612	2017	Brasil	Pará	São Félix do Xingu	Serra dos Carajás	Erva ca. 60 cm alt. Cálice verde; corola amarela com estrias castanhas; anteras amarelas.
Urticaceae	Pourouma	guianensis	Aubl.				Barbosa-Silva, R.G.	1308	2019	Brasil	Pará	São Félix do Xingu	Serra de Campos	Árvore 8 m alt. Folhas discolores. Inflorescência com raque verde. Frutos verde, cálice com tricomas ferrugíneos
Urticaceae	Cecropia	palmata	Willd.				Andrino, C.O.	664	2019	Brasil	Pará	São Félix do Xingu	Serra de Campos	Árvore ca. 10 m alt. Infrutescência verde
Velloziaceae	Vellozia	glauca	Pohl				Zappi, D.C.	3890	2018	Brasil	Pará	São Félix do Xingu	Serra de Campos, 20 km N de Tancredo Neves	Erva, frutos jovens verdes verrucosos
Velloziaceae	Vellozia	glauca	Pohl				Pastore, M.	641	2017	Brasil	Pará	São Félix do Xingu	Serra dos Carajás	Arbusto até 1,5 m alt. Frutos verdes.
Verbenaceae	Stachytarpheta	cayennensis	(Rich.) Vahl				Andrino, C.O.	608	2019	Brasil	Pará	São Félix do Xingu	Serra de Campos	Flores alvas com leve tom azulado.
Verbenaceae	Lippia	grata	Schauer				Zappi, D.C.	3871	2018	Brasil	Pará	São Félix do Xingu	Serra de Campos, 20 km N de Tancredo Neves	Arbusto 2 m alt.., folhas aromáticas, flores alvas com fauce amarelada
Verbenaceae	Lippia	grata	Schauer				Viana, P.L.	6114	0	Brasil	Pará	São Félix do Xingu	Serra de Campos	
Verbenaceae	Lippia	grata	Schauer				Nogueira, M.G.C.	726	2017	Brasil	Pará	São Félix do Xingu	Serra de Campos	Arbusto ramificado com cerca de 2 m de alt., folhas discolores com face superior verde e inferior acinzentada, perfumadas, flores brancas.
Verbenaceae	Lantana	trifolia	L.				Nogueira, M.G.C.	755	2017	Brasil	Pará	São Félix do Xingu	Vila Tancredo	Cálice verde com corola roxa.
Vitaceae	Cissus	erosa	Rich.				Zappi, D.C.	3882	2018	Brasil	Pará	São Félix do Xingu	Serra de Campos, 20 km N de Tancredo Neves	Trepadeira, inflorescências vermelhas
Vitaceae	Cissus	erosa	Rich.				Pastore, M.	617	2017	Brasil	Pará	São Félix do Xingu	Serra dos Carajás	Trepadeira com flores vermelhas e frutos verdes.
Vitaceae	Cissus	erosa	Rich.				Nogueira, M.G.C.	725	2017	Brasil	Pará	São Félix do Xingu	Serra de Campos	Trepadeira, inflorescência vermelha.
Vochysiaceae	Qualea	parviflora	Mart.				Falcão, B.F.	649	2016	Brasil	Pará	São Félix do Xingu	Serra da Seringa	Arbóreo. Frutos da cor marrom, dispersando.. Pto. E1SER11JUN.
Vochysiaceae	Qualea	parviflora	Mart.				Pastore, M.	624	2017	Brasil	Pará	São Félix do Xingu	Serra dos Carajás	Árvore até 3 m alt.. Folhas coriáceas e frutos imaturos com indumento.

Xyridaceae	Xyris	brachysepala	Kral	Viana, P.L.	6125	0	Brasil	Pará	São Félix do Xingu	Serra de Campos	
Xyridaceae	Xyris	brachysepala	Kral	Viana, P.L.	6137	0	Brasil	Pará	São Félix do Xingu	Serra de Campos	
Xyridaceae	Xyris	brachysepala	Kral	Falcão, B.F.	624	2016	Brasil	Pará	São Félix do Xingu	Serra da Seringa	Erva terrestre. Inflorescências ferrugíneas. Corola amarela.. Pto. E3SER02JUN.
Xyridaceae	Xyris	brachysepala	Kral	Falcão, B.F.	640	2016	Brasil	Pará	São Félix do Xingu	Serra da Seringa	Erva terrestre. Inflorescência marrom, pétalas amarelas. Associada a campos hidromórficos. . Pto. E1SER08JUN.
Xyridaceae	Xyris	brachysepala	Kral	Nogueira, M.G.C.	740	2017	Brasil	Pará	São Félix do Xingu	Serra de Campos	Erva com flores amarelas.
